# Crystal structure of a two-dimensional metal–organic framework assembled from lithium(I) and γ-cyclo­dextrin

**DOI:** 10.1107/S2056989020001942

**Published:** 2020-02-14

**Authors:** Kristine Krukle-Berzina, Sergey Belyakov, Anatoly Mishnev, Kirill Shubin

**Affiliations:** a Latvian Institute of Organic Synthesis, 21 Aizkraukles street, Riga, LV-1006, Latvia

**Keywords:** lithium, metal–organic framework, γ-cyclo­dextrin, crystal structure, SQUEEZE procedure

## Abstract

The crystal structure of the polymeric title compound, *catena*-poly[[[di­aqua­lithium]-μ-γ-cyclo­dextrin(1−)-[aqua­lithium]-μ-γ-cyclo­dextrin(1−)] pentadecahydrate], {[Li_2_(C_48_H_79_O_40_)_2_(H_2_O)_3_]·15H_2_O}_*n*_, consists of deprotonated γ-cyclo­dextrin (CD) mol­ecules assembled by lithium ions into metal–organic ribbons that are cross-linked by multiple O—H⋯O hydrogen bonds into sheets extending parallel to (01). Within a ribbon, one Li^+^ ion is coordinated by one deprotonated hydroxyl group of the first γ-CD torus and by one hydroxyl group of the second γ-CD torus as well as by two water mol­ecules. The other Li^+^ ion is coordinated by one deprotonated hydroxyl and by one hydroxyl group of the second γ-CD torus, by one hydroxyl group of the first γ-CD torus as well as by one water mol­ecule. The coordination spheres of both Li^+^ cations are distorted tetra­hedral. The packing of the structure constitute channels along the *a* axis. Parts of the hy­droxy­methyl groups in cyclo­dextrin molecules as well as water mol­ecules show two-component disorder. Electron density associated with additional disordered solvent mol­ecules inside the cavities was removed with the SQUEEZE [Spek (2015 ▸). *Acta Cryst.* C**71**, 9–18] routine in *PLATON*. These solvent mol­ecules are not considered in the given chemical formula and other crystal data. Five out of the sixteen hy­droxy­methyl groups and one water mol­ecule are disordered over two sets of sites.

## Abstract 2

The first metal–organic framework (MOF) formed from lithium(I) and γ-cyclo­dextrin is reported. The structure is characterized by an unusually low metal/ligand ratio.

## Chemical context   

Metal–organic frameworks (MOFs) based on cyclo­dextrin were developed by the Stoddart group and have been known for almost ten years (Smaldone *et al.*, 2010 ▸). Many cyclo­dextrin MOFs with various alkali metal ions have been obtained so far (Patel *et al.*, 2017 ▸; Bagabas *et al.*, 2013 ▸). Exceptions are lithium ion-based MOFs because all of the compounds obtained that have been reported in the literature contain two different metal ions in the crystal structure (Bagabas *et al.*, 2013 ▸; Patel *et al.*, 2017 ▸). Lithium-based MOFs are among the best candidates for electrode materials for lithium–ion batteries because of their high porosity and structural control (Baumann *et al.*, 2019 ▸; Sharma *et al.*, 2019 ▸). Another potential application of lithium–cyclo­dextrin MOFs is based on their excellent biocompatibility and low toxicity. Analogous materials with sodium and potassium ions have been studied in the pharmaceutical and biomedicine fields (Han *et al.*, 2018 ▸). In view of the importance of the properties of such MOFs, we have successfully synthesized the lithium-based title compound, and report herein its crystal structure.
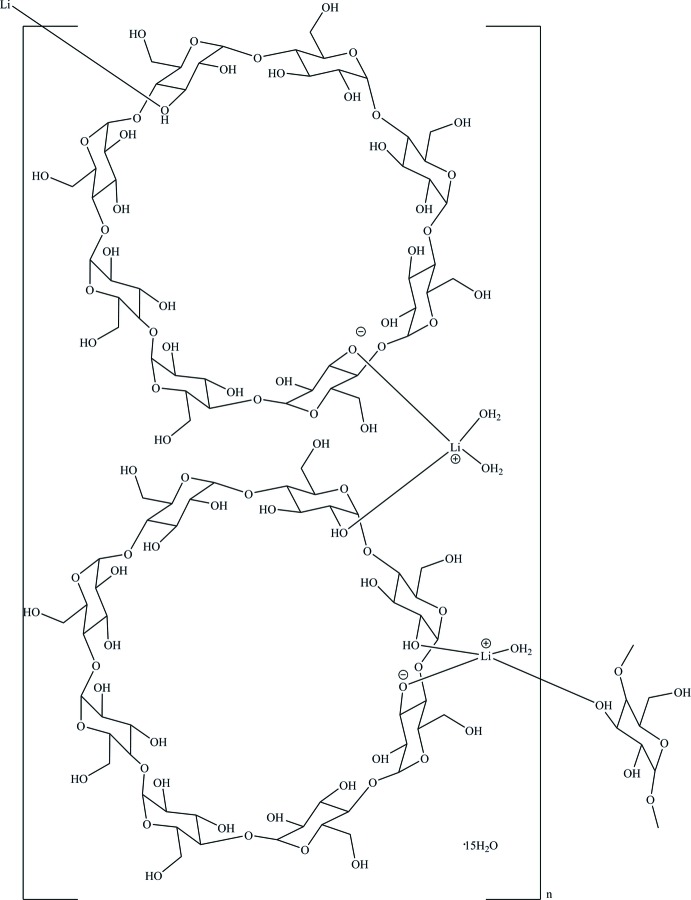


### Structural commentary   

**Figure 1:**
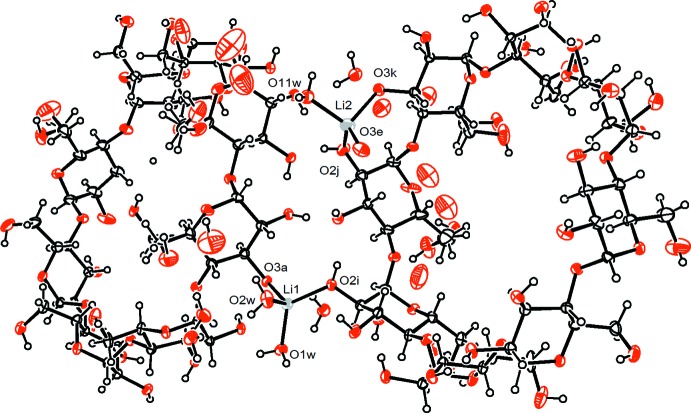
The asymmetric unit of the title compound drawn with displacement ellipsoids at the 50% probability level. Except for the two Li and coordinating O sites, atomic labels are not shown for clarity.

The structure comprises of two deprotonated γ-cyclo­dextrin (γ-CD) molecules, two lithium cations and eighteen water mol­ecules in the asymmetric unit (Fig. 1 ▸). To distinguish between the first and the second γ-CD molecule, we have assigned the names CD-AH and CD-IP, respectively, and have defined the side of the γ-CD toroid containing the hy­droxy­methyl groups as the ‘top’ and the opposite side, having hydroxyl groups, as the ‘bottom’. Other details of the labelling scheme used are given in the *Refinement* section.

Both Li^+^ ions are coordinated by four oxygen atoms in the form of distorted tetra­hedra. The Li1^+^ cation is bonded to an oxygen atom of a deprotonated hydroxyl group belonging to the first *γ*-CD torus [Li1—O3*A* = 1.977 (6) Å], to an oxygen atom of a hydroxyl group belonging to the second γ-CD molecule through a dative bond [Li1—O2*I* = 1.921 (6) Å], and to two water mol­ecules [Li1—O1*W* = 1.908 (6) Å, Li1—O2*W* = 1.882 (6) Å]. The Li2^+^ ion is bonded to one deprotonated hydroxyl oxygen atom and to one hydroxyl oxygen atom of the second *γ*-CD torus [Li2—O3*K* = 1.979 (7) Å and Li2—O2*J* = 1.902 (8) Å, respectively], to a hydroxyl oxygen atom of the first *γ*-CD torus of another unit cell [Li2—O3*E*(*x*, *y* + 1, *z* + 1) = 1.973 (8) Å], as well as to one water mol­ecule [Li2—O11*W* = 1.954 (8) Å]. All hydroxyl groups in a CD-AH fragment form intra­molecular O—H⋯O hydrogen bonds of medium strength between adjacent glucose units around the bottom of the *γ*-CD torus (Table 1 ▸). In a CD-IP fragment, oxygen atoms O3*K* and O2*J* do not participate in intra­molecular hydrogen bonding but coordinate to the Li2^+^ cation. Five out of the sixteen hy­droxy­methyl groups (in the *A*, *E*, *H*, *J* and *K* glucose units) and one water mol­ecule are disordered over two sets of sites.

## Supra­molecular features   

**Figure 2:**
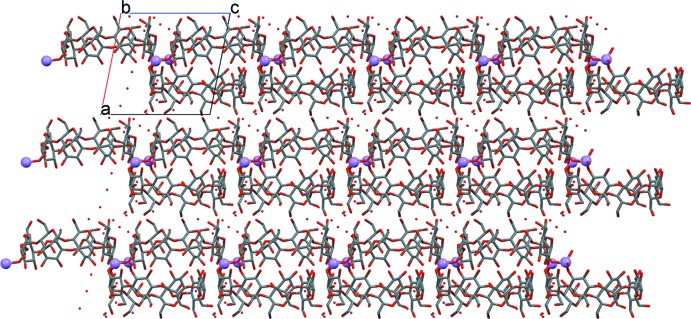
Ribbons of γ-CD tori and lithium ions consolidated by O—H⋯O hydrogen bonds (not shown) into sheets extending parallel to (0
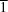
1).

In the crystal structure, the deprotonated γ-CD molecules are linked by the lithium cations into infinite ribbons (Fig. 2 ▸) running parallel to [011] and consolidated by O—H⋯O hydrogen bonds into sheets extending parallel to (0
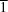
1). Therefore the crystal structure can be described as that of a two-dimensional MOF. It should be noted that in the asymmetric unit, the top and bottom of the CD-IP torus have inverted positions relative to the top and bottom of the CD-AH torus. The crystal packing shows that in the sheets there are additional ‘bottom-to-bottom’ inter­molecular hydrogen-bonding inter­actions between adjacent tori. However, not all the hydroxyl groups participate in these inter­actions. For instance, oxygen atoms O2*C*, O3*F*, O2*H* and O2*K* do not form inter­molecular hydrogen bonds. On the other hand, oxygen atom O2*G* takes part in two inter­molecular hydrogen bonds. On the whole, the strength of the hydrogen bonds in ‘bottom-to-bottom’ inter­actions are moderate to weak since most of these bonds are bifurcated, giving rise to both intra- and inter­molecular bonds. The ribbons formed by the lithium cations and γ-CD molecules are mainly assembled into sheets by means of ‘top-to-top’ inter­molecular hydrogen-bonding inter­actions between adjacent tori. In the ‘top-to-top’ inter­actions it is possible to distinguish three direct hydrogen bonds of moderate strength (O6*I*—H148⋯O6*C*, O6*P*—H152⋯O6*B*, O6*G*—H65⋯O6*M*, Table 1 ▸), two inter­actions by means of water mol­ecules O5*W* and O7*W*, and one inter­action through two water mol­ecules, O7*W* and O8*W*. Adjacent sheets are inter­connected through additional O—H⋯O hydrogen bonds, involving mainly water mol­ecules lying at the outsides of the sheets, *e.g*. O4*W*, O9*W*, O10*W*.

**Figure 3:**
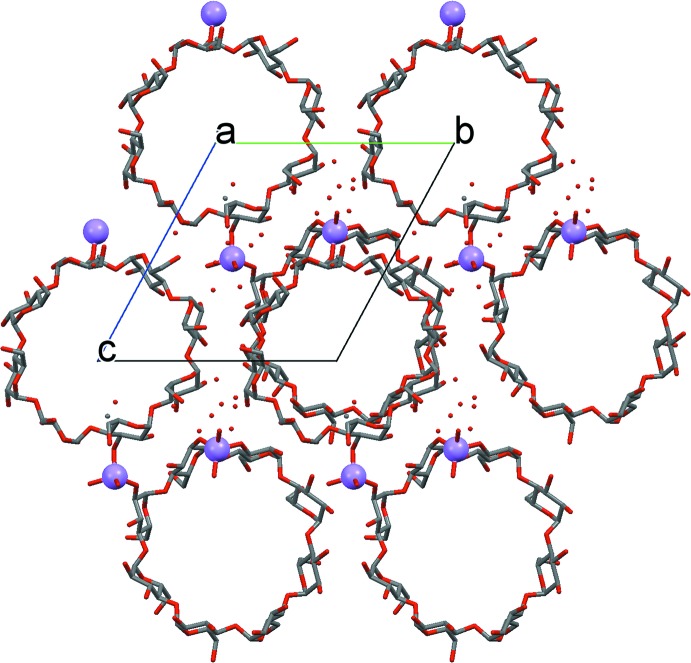
Channels formed by *γ*-CD rings along the *a* axis.

A remarkable feature of the crystal packing is the formation of channels along the *a* axis (Fig. 3 ▸). These channels are filled with disordered solvent mol­ecules that could not be modelled on basis of the current diffraction data (see *Refinement* section for details).

## Database survey   

A search in the Cambridge Structural Database (CSD, Version 5.40, update November 2018; Groom *et al.*, 2016 ▸) revealed 15 entries containing cyclo­dextrin moieties with lithium cations. The number of entries with cyclo­dextrin derivatives that contain solely lithium as a metal ion is two, *viz*. CYDXLI10 (Noltemeyer & Saenger, 1980 ▸) and FEJFIJ (Kamitori *et al.*, 1987 ▸). However, in both cases they do not form a polymeric coordination compound. There are also two metal–organic frameworks built on coordination of lithium cations, but in each case lithium is assisted by another metal, *viz*. manganese in FEVPEC (Geisselmann *et al.*, 2005 ▸) and copper in YAPKOP (Fuchs *et al.*, 1993 ▸). All other crystal structures containing lithium and cyclo­dextrin also contain a transition or a main group metal that forms metal–organic frameworks or dimers.

## Synthesis and crystallization   

All solvents and chemicals were obtained from commercial sources and were used without additional purification. The synthetic procedure was analogous to that reported for the sodium compound (Newton *et al.*, 2016 ▸). Oxidovanadium(IV) sulfate hydrate (55 mg, 0.25 mmol) and γ–cyclo­dextrin (70 mg, 0.054 mmol) were suspended in water (1.0 ml). Lithium hydroxide (31 mg, 1.29 mmol) and γ–cyclo­dextrin (70 mg, 0.054 mmol) were dissolved in water (0.5 ml) and added to the suspension. After stirring for several minutes, the solid oxidovanadium(IV) sulfate dissolved to yield a green solution. The flask containing this solution was placed into a sealable container filled with acetone, and crystals were obtained by the vapour diffusion method. The precipitate contained crystals of two different forms. Whereas the large colourless cuboid crystals were not suitable for X-ray diffraction studies since their diffraction intensities were limited to 2 Å, the smaller plate-like colourless crystals were of good quality and were subjected to single-crystal X-ray diffraction analysis.

## Refinement   

Crystal data, data collection and structure refinement details are summarized in Table 2 ▸. The atom numbering scheme is as follows: The atoms in the d-gluco­pyran­oside units are numbered according to the rules for sugars, and a suffix from *A* to *P* is added at the end of the label to distinguish sixteen different glucose units. Labels of water mol­ecules are marked with a letter *W* at the end.

Several disordered atomic fragments and solvent mol­ecules, as well as a large number of water mol­ecules are present in the crystal structure. To make the refinement stable, it was necessary to apply restraints for the bond lengths (DFIX, SADI), bond angles (DANG), and displacement parameters (SIMU, ISOR) of the disordered moieties. Hydrogen-atom positions of the hydroxyl groups were calculated geometrically and refined using the riding-model approximation, with *U*_iso_(H) = 1.5*U*_eq_(O). Five out of sixteen hy­droxy­methyl groups in the two *γ*-CD moieties were found to be disordered over two sets of sites. Eighteen oxygen atoms belonging to water mol­ecules were localized from difference-Fourier maps in the space outside the lithium *γ*-CD ribbons. Water oxygen atoms O12*W* and O13*W* represent two-component positional disorder of a water mol­ecule with refined occupancy factors of 0.578 (12) and 0.422 (12), respectively. Hydrogen atoms were reliably assigned for only eleven of the water mol­ecules. For the other water mol­ecules, modelling of hydrogen atoms lead to unstable refinements, and therefore these oxygen atoms were left as isolated.

Electron density associated with additional disordered solvent mol­ecules inside the cavities was removed by means of the *SQUEEZE* procedure of *PLATON* program (Spek, 2015 ▸). The solvent-accessible volume is 845 Å^3^, the number of electrons in the cavities being 237. Since the solvent did not contain exclusively water but was a mixture of water and acetone, it was not possible to determine its content from these numbers. Therefore the chemical formula and crystal data given in Table 2 ▸ do not take into account these solvent mol­ecules.

## Supplementary Material

Crystal structure: contains datablock(s) I. DOI: 10.1107/S2056989020001942/wm5534sup1.cif

Structure factors: contains datablock(s) I. DOI: 10.1107/S2056989020001942/wm5534Isup2.hkl

Supporting information file. DOI: 10.1107/S2056989020001942/wm5534Isup3.cdx

CCDC reference: 1983285

Additional supporting information:  crystallographic information; 3D view; checkCIF report

## Figures and Tables

**Table 1:** Hydrogen-bond geometry (Å, °)

*D*—H⋯*A*	*D*—H	H⋯*A*	*D*⋯*A*	*D*—H⋯*A*
O2*K*—H171⋯O3*B* ^i^	0.82	1.89	2.688 (3)	165
O2*P*—H154⋯O3*I*	0.82	2.07	2.799 (4)	148
O3*B*—H79⋯O2*C*	0.82	2.20	2.698 (3)	119
O3*C*—H83⋯O3*P* ^ii^	0.82	1.99	2.774 (3)	161
O3*L*—H170⋯O2*K*	0.82	2.04	2.788 (3)	152
O3*H*—H70⋯O2*A*	0.82	2.07	2.533 (3)	116
O3*O*—H157⋯O2*N*	0.82	2.11	2.915 (3)	168
O2*O*—H158⋯O3*B* ^iii^	0.82	1.64	2.450 (3)	167
O3*G*—H67⋯O2*P* ^iv^	0.82	2.00	2.714 (3)	146
O3*D*—H88⋯O2*E*	0.82	2.15	2.702 (4)	125
O2*D*—H87⋯O3*C*	0.82	2.05	2.819 (3)	156
O3*M*—H165⋯O3*G* ^iii^	0.82	2.04	2.820 (3)	160
O6*G*—H65⋯O6*M* ^v^	0.82	1.87	2.636 (4)	154
O3*P*—H153⋯O2*O*	0.82	1.82	2.611 (3)	163
O2*B*—H80⋯O3*O* ^ii^	0.82	1.98	2.772 (3)	161
O2*L*—H169⋯O3*M*	0.82	2.15	2.888 (4)	149
O2*C*—H84⋯O3*W* ^vi^	0.82	2.42	3.116 (4)	144
O1*W*—H18*B*⋯O2*G* ^vii^	0.86	1.93	2.781 (4)	169
O2*H*—H71⋯O3*H*	0.82	2.38	2.813 (3)	113
O6*I*—H148⋯O6*C* ^viii^	0.82	1.95	2.735 (4)	161
O2*I*—H149⋯O4*J*	0.82	2.43	2.818 (3)	110
O2*I*—H149⋯O3*J*	0.82	1.92	2.689 (4)	156
O3*J*—H176⋯O2*E* ^iii^	0.82	1.78	2.570 (4)	161
O2*A*—H73⋯O3*J*	0.82	1.64	2.448 (3)	167
O2*N*—H162⋯O2*A* ^iii^	0.82	1.96	2.768 (3)	170
O6*L*—H168⋯O7*W* ^ix^	0.82	1.98	2.771 (4)	161
O6*C*—H82⋯O17*W* ^vi^	0.82	2.18	2.838 (6)	137
O3*I*—H150⋯O3*D* ^iii^	0.82	2.03	2.817 (3)	160
O6*F*—H61⋯O5*B* ^iv^	0.82	1.93	2.674 (3)	151
O2*G*—H66⋯O3*F*	0.82	2.17	2.883 (5)	146
O11*W*—H18*C*⋯O2*C* ^i^	0.87	1.96	2.753 (4)	151
O11*W*—H18*D*⋯O16*W*	0.86	2.35	2.793 (9)	112
O2*W*—H17*A*⋯O8*W*	0.87	2.03	2.723 (4)	135
O2*W*—H17*B*⋯O10*W*	0.87	2.53	3.306 (9)	149
O2*J*—H174⋯O3*H*	0.86 (1)	1.85 (1)	2.677 (3)	160 (2)
O2*M*—H166⋯O3*N*	0.82	2.10	2.852 (4)	153
O6*B*—H78⋯O9*W* ^x^	0.82	1.93	2.732 (4)	164
O3*E*—H59⋯O2*F*	0.82	2.04	2.705 (4)	137
O2*E*—H57⋯O3*J* ^ii^	0.82	1.77	2.570 (4)	163
O6*D*—H86⋯O5*D*	0.82	2.38	2.789 (4)	112
O6*D*—H86⋯O5*W* ^v^	0.82	2.15	2.767 (5)	132
O6*P*—H152⋯O6*B* ^viii^	0.82	1.89	2.710 (4)	179
O6*H*1—H69*A*⋯O17*W*	0.82	2.49	3.229 (14)	150
O6*H*2—H69*B*⋯O6*N* ^v^	0.82	2.32	2.969 (9)	137
O6*N*—H160⋯O14*W* ^iii^	0.82	1.97	2.714 (10)	151
O6*A*2—H76*B*⋯O14*W* ^xi^	1.19	2.11	3.276 (14)	166
O6*A*1—H76*A*⋯O6*N* ^v^	0.82	1.94	2.755 (7)	170

Symmetry codes: (i) 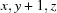; (ii) 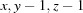; (iii) 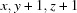; (iv) 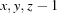; (v) 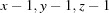; (vi) 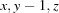; (vii) 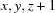; (viii) 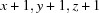; (ix) 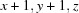; (x) 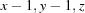; (xi) 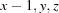.

**Table 2:** Experimental details

Crystal data	
Chemical formula	[Li_2_(C_48_H_79_O_40_)_2_(H_2_O)_3_]·15H_2_O
*M*_r_	2930.26
Crystal system, space group	Triclinic, *P*1
Temperature (K)	160
*a*, *b*, *c* (Å)	15.00386 (18), 17.0413 (2), 17.64915 (15)
α, β, γ (°)	117.0411 (10), 96.8906 (9), 96.8281 (10)
*V* (Å^3^)	3912.77 (8)
*Z*	1
Radiation type	Cu *K*α
μ (mm^−1^)	0.99
Crystal size (mm)	0.20 × 0.12 × 0.06

Data collection	
Diffractometer	XtaLAB Synergy, Dualflex, HyPix
Absorption correction	Multi-scan (*CrysAlis PRO*; Rigaku OD, 2018 ▸)
*T*_min_, *T*_max_	0.882, 1.000
No. of measured, independent and observed [*I* > 2σ(*I*)] reflections	73131, 24072, 22828
*R*_int_	0.040
(sin θ/λ)_max_ (Å^−1^)	0.631

Refinement	
*R*[*F* ^2^ > 2σ(*F* ^2^)], *wR*(*F* ^2^), *S*	0.046, 0.133, 1.04
No. of reflections	24072
No. of parameters	1881
No. of restraints	1760
H-atom treatment	H atoms treated by a mixture of independent and constrained refinement
Δρ_max_, Δρ_min_ (e Å^−3^)	0.52, −0.44
Absolute structure	Flack *x* determined using 7110 quotients [(*I* ^+^)−(*I* ^−^)]/[(*I* ^+^)+(*I* ^−^)] (Parsons *et al.*, 2013 ▸)
Absolute structure parameter	0.07 (6)

Computer programs: *CrysAlis PRO* (Rigaku OD, 2018 ▸), *SHELXT2014/4* (Sheldrick, 2015 ▸a), *OLEX2* (Dolomanov *et al.*, 2009 ▸), *Mercury* (Macrae *et al.*, 2020 ▸) and *publCIF* (Westrip, 2010 ▸).

## supplementary crystallographic information

### Crystal data

**Table:**

[Li_2_(C_48_H_79_O_40_)_2_(H_2_O)_3_]·15H_2_O	*Z* = 1
*M**_r_* = 2930.26	*F*(000) = 1545
Triclinic, *P*1	*D*_x_ = 1.237 Mg m^−^^3^
*a* = 15.00386 (18) Å	Cu *K*α radiation, λ = 1.54184 Å
*b* = 17.0413 (2) Å	Cell parameters from 50853 reflections
*c* = 17.64915 (15) Å	θ = 3.7–76.2°
α = 117.0411 (10)°	µ = 0.99 mm^−^^1^
β = 96.8906 (9)°	*T* = 160 K
γ = 96.8281 (10)°	Block, colourless
*V* = 3912.77 (8) Å^3^	0.20 × 0.12 × 0.06 mm

### Data collection

**Table:**

XtaLAB Synergy, Dualflex, HyPix diffractometer	22828 reflections with *I* > 2σ(*I*)
Radiation source: micro-focus sealed X-ray tube	*R*_int_ = 0.040
ω scans	θ_max_ = 76.6°, θ_min_ = 3.0°
Absorption correction: multi-scan (*CrysAlisPro*; Rigaku OD, 2018)	*h* = −18→18
*T*_min_ = 0.882, *T*_max_ = 1.000	*k* = −21→21
73131 measured reflections	*l* = −17→22
24072 independent reflections	

### Refinement

**Table:**

Refinement on *F*^2^	Hydrogen site location: mixed
Least-squares matrix: full	H atoms treated by a mixture of independent and constrained refinement
*R*[*F*^2^ > 2σ(*F*^2^)] = 0.046	*w* = 1/[σ^2^(*F*_o_^2^) + (0.0977*P*)^2^ + 0.4036*P*] where *P* = (*F*_o_^2^ + 2*F*_c_^2^)/3
*wR*(*F*^2^) = 0.133	(Δ/σ)_max_ = 0.005
*S* = 1.04	Δρ_max_ = 0.52 e Å^−^^3^
24072 reflections	Δρ_min_ = −0.44 e Å^−^^3^
1881 parameters	Absolute structure: Flack *x* determined using 7110 quotients [(*I*^+^)-(*I*^-^)]/[(*I*^+^)+(*I*^-^)] (Parsons *et al.*, 2013)
1760 restraints	Absolute structure parameter: 0.07 (6)

### Special details

**Table:**

Geometry. All esds (except the esd in the dihedral angle between two l.s. planes) are estimated using the full covariance matrix. The cell esds are taken into account individually in the estimation of esds in distances, angles and torsion angles; correlations between esds in cell parameters are only used when they are defined by crystal symmetry. An approximate (isotropic) treatment of cell esds is used for estimating esds involving l.s. planes.

### Fractional atomic coordinates and isotropic or equivalent isotropic displacement parameters (Å^2^)

**Table:**

	*x*	*y*	*z*	*U*_iso_*/*U*_eq_	Occ. (<1)
Li1	0.4576 (4)	0.3327 (4)	0.5304 (4)	0.0269 (10)	
Li2	0.4710 (5)	0.6996 (5)	0.4054 (5)	0.0458 (15)	
O4G	0.33893 (14)	0.36201 (14)	−0.02301 (14)	0.0227 (4)	
O4B	0.25943 (14)	−0.16976 (13)	0.18179 (14)	0.0231 (4)	
O4I	0.69022 (14)	0.63042 (14)	0.86918 (15)	0.0250 (4)	
O5L	0.88522 (13)	1.24020 (14)	0.70450 (15)	0.0257 (4)	
O4M	0.77825 (14)	1.26949 (14)	0.79513 (14)	0.0233 (4)	
O5D	0.13849 (14)	−0.42259 (16)	−0.22411 (15)	0.0273 (4)	
O4C	0.24639 (14)	−0.35567 (14)	−0.09445 (14)	0.0241 (4)	
O4L	0.74348 (15)	1.02740 (13)	0.53319 (14)	0.0248 (4)	
O2K	0.57505 (14)	0.97489 (14)	0.42374 (15)	0.0265 (4)	
H171	0.528720	0.950440	0.385696	0.040*	
O5B	0.22827 (13)	0.03758 (13)	0.36190 (14)	0.0235 (4)	
O4P	0.69882 (13)	0.88077 (13)	1.11826 (14)	0.0228 (4)	
O2P	0.54964 (15)	0.57295 (15)	0.93057 (16)	0.0295 (5)	
H154	0.546770	0.573920	0.884320	0.044*	
O3B	0.44096 (13)	−0.09424 (14)	0.28231 (15)	0.0239 (4)	
H79	0.423187	−0.131089	0.297725	0.036*	
O3C	0.38207 (13)	−0.37652 (14)	0.01378 (15)	0.0251 (4)	
H83	0.434846	−0.350973	0.039096	0.038*	
O5C	0.14806 (13)	−0.29665 (15)	0.10169 (14)	0.0267 (4)	
O4H	0.31547 (15)	0.33194 (14)	0.21361 (15)	0.0268 (4)	
O4F	0.34970 (16)	0.16664 (16)	−0.28587 (15)	0.0303 (5)	
O4A	0.30249 (14)	0.12114 (14)	0.30616 (15)	0.0245 (4)	
O3L	0.63979 (14)	1.15876 (14)	0.52991 (15)	0.0266 (4)	
H170	0.606220	1.109199	0.508305	0.040*	
O5O	0.78269 (14)	0.99495 (14)	1.25126 (15)	0.0250 (4)	
O5K	0.80409 (14)	0.92478 (14)	0.42205 (17)	0.0296 (5)	
O4J	0.69125 (16)	0.57746 (15)	0.59780 (16)	0.0301 (5)	
O3A	0.45578 (14)	0.26648 (14)	0.40452 (15)	0.0256 (4)	
O3H	0.48013 (13)	0.43926 (15)	0.22488 (14)	0.0246 (4)	
H70	0.493145	0.396666	0.230719	0.037*	
O3O	0.54312 (14)	1.08832 (15)	1.22884 (17)	0.0290 (5)	
H157	0.555526	1.141159	1.240219	0.044*	
O2O	0.53771 (14)	0.90287 (14)	1.17899 (16)	0.0286 (5)	
H158	0.500860	0.907893	1.210907	0.043*	
O3G	0.49582 (14)	0.41048 (15)	−0.07879 (15)	0.0277 (4)	
H67	0.488399	0.457056	−0.079508	0.042*	
O4K	0.69440 (17)	0.73763 (15)	0.45141 (15)	0.0305 (5)	
O5H	0.23991 (15)	0.42572 (18)	0.07078 (16)	0.0324 (5)	
O3D	0.38241 (15)	−0.45261 (15)	−0.31235 (15)	0.0270 (4)	
H88	0.362129	−0.467210	−0.363160	0.040*	
O2D	0.35733 (16)	−0.47881 (15)	−0.16802 (15)	0.0294 (5)	
H87	0.366213	−0.436692	−0.118678	0.044*	
O5P	0.79307 (14)	0.67996 (15)	0.99836 (16)	0.0307 (5)	
O4O	0.70942 (14)	1.17643 (14)	1.21559 (14)	0.0247 (4)	
O3M	0.66831 (16)	1.39076 (17)	0.87463 (16)	0.0325 (5)	
H165	0.625494	1.409037	0.897862	0.049*	
O6G	0.14077 (16)	0.37150 (17)	−0.15454 (17)	0.0332 (5)	
H65	0.102961	0.391534	−0.124551	0.050*	
O5I	0.78573 (15)	0.52567 (15)	0.67182 (15)	0.0283 (4)	
O3P	0.55256 (14)	0.73672 (14)	1.08289 (16)	0.0280 (5)	
H153	0.558371	0.789660	1.118925	0.042*	
O2B	0.47351 (13)	0.10068 (14)	0.37156 (16)	0.0250 (4)	
H80	0.4858 (8)	0.0854 (10)	0.323 (3)	0.037*	
O4N	0.76379 (15)	1.32522 (15)	1.07547 (15)	0.0270 (4)	
O4D	0.27014 (15)	−0.34672 (15)	−0.34911 (15)	0.0276 (4)	
O2L	0.67061 (16)	1.31983 (15)	0.69207 (16)	0.0308 (5)	
H169	0.662053	1.319167	0.736814	0.046*	
O5N	0.81545 (16)	1.31182 (16)	1.27765 (16)	0.0320 (5)	
O5A	0.22133 (15)	0.31348 (16)	0.30186 (18)	0.0318 (5)	
O2C	0.38417 (15)	−0.27426 (15)	0.20083 (15)	0.0279 (4)	
H84	0.419054	−0.309855	0.189874	0.042*	
O1W	0.48557 (15)	0.24468 (16)	0.56322 (16)	0.0303 (5)	
H18A	0.445451	0.195735	0.534311	0.045*	
H18B	0.480260	0.261930	0.616067	0.045*	
O5M	0.88729 (15)	1.36866 (19)	1.02618 (16)	0.0336 (5)	
O2H	0.48761 (15)	0.49678 (16)	0.09911 (16)	0.0315 (5)	
H71	0.529032	0.486102	0.125594	0.047*	
O5G	0.25089 (15)	0.26615 (15)	−0.25711 (15)	0.0276 (4)	
O6I	0.88926 (16)	0.52360 (17)	0.80971 (17)	0.0339 (5)	
H148	0.924438	0.534406	0.854085	0.051*	
O4E	0.32910 (18)	−0.13183 (17)	−0.42103 (18)	0.0362 (5)	
O2I	0.54292 (17)	0.44324 (17)	0.57023 (17)	0.0365 (6)	
H149	0.540797	0.483110	0.556604	0.055*	
O3J	0.53090 (15)	0.53372 (16)	0.47931 (16)	0.0310 (5)	
H176	0.493507	0.562976	0.503116	0.046*	
O5F	0.2875 (2)	−0.04718 (16)	−0.48551 (17)	0.0374 (5)	
O2A	0.46924 (15)	0.39526 (15)	0.34311 (16)	0.0292 (5)	
H73	0.496848	0.438267	0.389003	0.044*	
O2N	0.56607 (17)	1.26861 (17)	1.24669 (19)	0.0364 (6)	
H162	0.535860	1.306944	1.270032	0.055*	
O6L	0.98554 (17)	1.14392 (19)	0.58507 (19)	0.0422 (6)	
H168	1.000376	1.198653	0.614766	0.063*	
O6C	0.03504 (18)	−0.42468 (19)	−0.05763 (19)	0.0417 (6)	
H82	0.062580	−0.435328	−0.021402	0.063*	
O3K	0.54173 (15)	0.78671 (16)	0.37916 (18)	0.0340 (5)	
O3I	0.53883 (15)	0.5026 (2)	0.75155 (17)	0.0378 (6)	
H150	0.489736	0.502015	0.725088	0.057*	
O6F	0.19948 (19)	0.10289 (17)	−0.47574 (17)	0.0372 (5)	
H61	0.198261	0.066781	−0.526437	0.056*	
O2G	0.49327 (18)	0.3003 (2)	−0.26120 (18)	0.0458 (7)	
H66	0.507964	0.260180	−0.251868	0.069*	
O11W	0.3588 (2)	0.6672 (2)	0.3201 (2)	0.0454 (6)	
H18C	0.360749	0.701641	0.296106	0.068*	
H18D	0.312791	0.678308	0.345859	0.068*	
O2W	0.34872 (17)	0.36492 (18)	0.5676 (2)	0.0428 (6)	
H17A	0.316253	0.322075	0.572224	0.064*	
H17B	0.313053	0.370150	0.528180	0.064*	
O2J	0.53701 (18)	0.60521 (18)	0.35856 (18)	0.0383 (6)	
H174	0.507 (2)	0.5568 (16)	0.314 (2)	0.057*	
O6O	0.88795 (19)	1.15883 (18)	1.37228 (19)	0.0443 (6)	
H156	0.837298	1.146368	1.381538	0.066*	
O6M	1.00303 (18)	1.3825 (2)	0.9272 (2)	0.0441 (6)	
H164	0.983089	1.392302	0.887631	0.066*	
O3N	0.59929 (18)	1.3443 (2)	1.1292 (2)	0.0422 (6)	
H161	0.613786	1.399001	1.159313	0.063*	
O2M	0.67426 (19)	1.45197 (18)	1.05828 (18)	0.0375 (5)	
H166	0.646448	1.409028	1.060761	0.056*	
O6B	0.03985 (17)	−0.04866 (19)	0.27087 (19)	0.0429 (6)	
H78	0.026222	−0.020508	0.318321	0.064*	
O3F	0.52019 (18)	0.1204 (2)	−0.3083 (2)	0.0479 (7)	
H63	0.549781	0.116408	−0.268435	0.072*	
O5J	0.7782 (2)	0.6269 (3)	0.4357 (3)	0.0651 (11)	
O3E	0.4539 (2)	−0.2536 (2)	−0.4736 (2)	0.0460 (7)	
H59	0.490001	−0.207565	−0.438177	0.069*	
O5E	0.1882 (2)	−0.3440 (2)	−0.4677 (2)	0.0504 (7)	
O2E	0.4107 (2)	−0.3985 (2)	−0.4311 (2)	0.0553 (9)	
H57	0.439976	−0.426339	−0.467581	0.083*	
O6D	0.0396 (2)	−0.4458 (3)	−0.3795 (2)	0.0588 (9)	
H86	0.047627	−0.484547	−0.365459	0.088*	
O2F	0.5116 (2)	−0.0741 (2)	−0.4046 (2)	0.0522 (8)	
H62	0.561956	−0.040660	−0.382332	0.078*	
O6P	0.9038 (2)	0.8142 (2)	1.1544 (3)	0.0682 (11)	
H152	0.944959	0.855410	1.190119	0.102*	
C3O	0.61962 (19)	1.04633 (19)	1.2026 (2)	0.0228 (6)	
H128	0.613993	1.021832	1.139690	0.027*	
C2L	0.7403 (2)	1.27180 (19)	0.6609 (2)	0.0230 (6)	
H108	0.766822	1.294111	0.624655	0.028*	
C1K	0.73704 (19)	0.97918 (19)	0.4432 (2)	0.0226 (5)	
H98	0.745669	1.021595	0.420243	0.027*	
C4B	0.27773 (19)	−0.09871 (19)	0.2692 (2)	0.0229 (6)	
H18	0.278765	−0.123363	0.309755	0.027*	
C1L	0.81674 (19)	1.28998 (19)	0.7354 (2)	0.0238 (6)	
H109	0.844539	1.354072	0.764838	0.029*	
C1B	0.31065 (19)	0.09432 (19)	0.3709 (2)	0.0221 (5)	
H15	0.323037	0.147801	0.428007	0.027*	
C5I	0.7821 (2)	0.5915 (2)	0.7576 (2)	0.0259 (6)	
H140	0.775300	0.647941	0.757464	0.031*	
C1H	0.3231 (2)	0.4412 (2)	0.0453 (2)	0.0251 (6)	
H29	0.320691	0.486495	0.025852	0.030*	
C2K	0.64193 (19)	0.91997 (19)	0.4022 (2)	0.0214 (5)	
H99	0.636475	0.890830	0.339037	0.026*	
C3M	0.7196 (2)	1.3567 (2)	0.9220 (2)	0.0244 (6)	
H114	0.683208	1.301990	0.916478	0.029*	
C4K	0.7061 (2)	0.7958 (2)	0.4132 (2)	0.0239 (6)	
H101	0.702809	0.759715	0.350709	0.029*	
C4L	0.78089 (19)	1.12283 (18)	0.5763 (2)	0.0218 (5)	
H106	0.807754	1.139232	0.536315	0.026*	
C4C	0.23020 (19)	−0.3551 (2)	−0.0157 (2)	0.0227 (5)	
H11	0.207640	−0.416867	−0.027815	0.027*	
C1I	0.7067 (2)	0.5024 (2)	0.6088 (2)	0.0271 (6)	
H144	0.715164	0.454802	0.553536	0.033*	
C5L	0.85443 (19)	1.1452 (2)	0.6548 (2)	0.0256 (6)	
H105	0.828687	1.122352	0.691108	0.031*	
C3L	0.70319 (19)	1.1718 (2)	0.6043 (2)	0.0226 (5)	
H107	0.671099	1.148489	0.637340	0.027*	
C1D	0.2073 (2)	−0.4362 (2)	−0.1723 (2)	0.0238 (6)	
H1	0.180193	−0.481154	−0.157237	0.029*	
C5B	0.20300 (19)	−0.04334 (19)	0.2795 (2)	0.0237 (6)	
H19	0.195701	−0.027549	0.232522	0.028*	
C4O	0.7089 (2)	1.1130 (2)	1.2481 (2)	0.0234 (6)	
H129	0.711035	1.143921	1.310759	0.028*	
C3B	0.37231 (18)	−0.04419 (19)	0.2819 (2)	0.0214 (5)	
H17	0.372839	−0.031788	0.232907	0.026*	
C3K	0.6275 (2)	0.8467 (2)	0.4285 (2)	0.0252 (6)	
H100	0.625046	0.874316	0.490341	0.030*	
C4I	0.70031 (19)	0.55971 (19)	0.7878 (2)	0.0229 (6)	
H141	0.709375	0.506752	0.794026	0.028*	
C4H	0.3141 (2)	0.3953 (2)	0.1809 (2)	0.0257 (6)	
H32	0.306738	0.452989	0.226943	0.031*	
C5O	0.7905 (2)	1.0670 (2)	1.2293 (2)	0.0265 (6)	
H130	0.791898	1.042324	1.167403	0.032*	
C4P	0.71111 (19)	0.78935 (19)	1.0858 (2)	0.0238 (6)	
H136	0.729405	0.778077	1.134567	0.029*	
C5C	0.1591 (2)	−0.2983 (2)	0.0206 (2)	0.0267 (6)	
H12	0.180653	−0.236935	0.031289	0.032*	
C2D	0.2840 (2)	−0.47043 (19)	−0.2210 (2)	0.0241 (6)	
H2	0.257984	−0.530641	−0.269203	0.029*	
C1P	0.7129 (2)	0.6185 (2)	0.9428 (2)	0.0251 (6)	
H133	0.723353	0.557237	0.923696	0.030*	
C2P	0.6321 (2)	0.63080 (19)	0.9890 (2)	0.0249 (6)	
H134	0.644516	0.613941	1.034821	0.030*	
C4N	0.7591 (2)	1.3419 (2)	1.1609 (2)	0.0260 (6)	
H122	0.771367	1.406958	1.199240	0.031*	
C3C	0.32148 (19)	−0.31751 (19)	0.0496 (2)	0.0225 (6)	
H10	0.346943	−0.257413	0.058965	0.027*	
C2C	0.3040 (2)	−0.31241 (19)	0.1342 (2)	0.0231 (5)	
H9	0.282243	−0.373720	0.123680	0.028*	
C3P	0.62033 (19)	0.72757 (19)	1.03117 (19)	0.0228 (6)	
H135	0.599670	0.743103	0.985788	0.027*	
C4D	0.2370 (2)	−0.4045 (2)	−0.3154 (2)	0.0250 (6)	
H4	0.208491	−0.464088	−0.363409	0.030*	
C3A	0.38758 (19)	0.2627 (2)	0.3374 (2)	0.0233 (6)	
H24	0.405646	0.230425	0.281307	0.028*	
C2H	0.4031 (2)	0.4758 (2)	0.1214 (2)	0.0262 (6)	
H30	0.392291	0.531174	0.168214	0.031*	
C5P	0.7847 (2)	0.7730 (2)	1.0311 (2)	0.0304 (7)	
H137	0.766818	0.784601	0.982437	0.037*	
C3G	0.4212 (2)	0.3396 (2)	−0.1359 (2)	0.0235 (6)	
H38	0.432668	0.284703	−0.134811	0.028*	
C4M	0.80454 (19)	1.3362 (2)	0.8849 (2)	0.0231 (6)	
H115	0.838944	1.391140	0.889194	0.028*	
C4G	0.33059 (19)	0.3565 (2)	−0.10779 (19)	0.0219 (5)	
H39	0.318045	0.412856	−0.104863	0.026*	
C6G	0.1600 (2)	0.2932 (2)	−0.1509 (2)	0.0306 (7)	
H64A	0.114053	0.241332	−0.192692	0.037*	
H64B	0.157577	0.300101	−0.093572	0.037*	
C2O	0.61608 (19)	0.9703 (2)	1.2249 (2)	0.0240 (6)	
H127	0.615319	0.995256	1.286970	0.029*	
C1C	0.22856 (19)	−0.2583 (2)	0.1655 (2)	0.0235 (6)	
H8	0.216141	−0.257705	0.218959	0.028*	
C3D	0.3182 (2)	−0.4117 (2)	−0.2602 (2)	0.0236 (6)	
H3	0.348207	−0.351760	−0.213604	0.028*	
C3I	0.6123 (2)	0.5374 (2)	0.7235 (2)	0.0289 (6)	
H142	0.600399	0.591750	0.721467	0.035*	
C2M	0.7496 (2)	1.4271 (2)	1.0178 (2)	0.0293 (6)	
H113	0.783755	1.481044	1.019952	0.035*	
C1N	0.7277 (2)	1.2688 (2)	1.2749 (2)	0.0263 (6)	
H119	0.723753	1.276642	1.332892	0.032*	
C5M	0.8643 (2)	1.3037 (3)	0.9366 (2)	0.0302 (7)	
H116	0.833673	1.245835	0.929040	0.036*	
C6I	0.8726 (2)	0.6056 (2)	0.8134 (2)	0.0308 (7)	
H14A	0.921239	0.629997	0.793962	0.037*	
H14B	0.873005	0.648742	0.873000	0.037*	
C2B	0.39096 (18)	0.04397 (19)	0.3638 (2)	0.0219 (5)	
H16	0.398659	0.031312	0.412801	0.026*	
C1O	0.70214 (19)	0.92891 (19)	1.2083 (2)	0.0231 (6)	
H126	0.701780	0.886321	1.231339	0.028*	
C3N	0.6614 (2)	1.3025 (2)	1.1592 (2)	0.0288 (6)	
H121	0.648631	1.238393	1.117193	0.035*	
C4J	0.6947 (2)	0.5696 (2)	0.5146 (2)	0.0310 (7)	
H91	0.698633	0.507985	0.474171	0.037*	
C3J	0.6079 (2)	0.5921 (2)	0.4833 (2)	0.0257 (6)	
H92	0.604779	0.653952	0.524109	0.031*	
C4A	0.2950 (2)	0.2133 (2)	0.3345 (2)	0.0255 (6)	
H25	0.280352	0.239391	0.392631	0.031*	
C2N	0.6542 (2)	1.3111 (2)	1.2472 (2)	0.0300 (7)	
H120	0.664651	1.374977	1.289516	0.036*	
C2J	0.6147 (2)	0.5861 (2)	0.3953 (2)	0.0313 (7)	
H93	0.618809	0.524089	0.355985	0.038*	
C4F	0.3592 (2)	0.0951 (2)	−0.3650 (2)	0.0311 (7)	
H44	0.374356	0.118033	−0.404705	0.037*	
C2I	0.6227 (2)	0.4675 (2)	0.6342 (2)	0.0277 (6)	
H143	0.632924	0.413489	0.637786	0.033*	
C5H	0.2360 (2)	0.3621 (3)	0.1036 (2)	0.0317 (7)	
H33	0.242856	0.303878	0.058066	0.038*	
C5G	0.2535 (2)	0.2785 (2)	−0.1706 (2)	0.0256 (6)	
H40	0.264847	0.223570	−0.169296	0.031*	
C6B	0.1118 (2)	−0.0930 (2)	0.2793 (3)	0.0360 (8)	
H77A	0.098975	−0.153822	0.231533	0.043*	
H77B	0.115229	−0.096473	0.332912	0.043*	
C1J	0.7003 (2)	0.6472 (2)	0.3999 (2)	0.0327 (7)	
H94	0.704803	0.636699	0.341229	0.039*	
C5D	0.1676 (2)	−0.3649 (2)	−0.2595 (2)	0.0301 (7)	
H5	0.195658	−0.304644	−0.212388	0.036*	
C6L	0.9402 (2)	1.1077 (2)	0.6311 (2)	0.0342 (7)	
H16A	0.981755	1.120867	0.683736	0.041*	
H16B	0.923400	1.042913	0.595940	0.041*	
C1F	0.3553 (3)	−0.0918 (2)	−0.4711 (3)	0.0370 (8)	
H47	0.362716	−0.138794	−0.527239	0.044*	
C5K	0.7987 (2)	0.8606 (2)	0.4547 (3)	0.0311 (7)	
H102	0.810724	0.889468	0.518251	0.037*	
C6F	0.1866 (2)	0.0560 (2)	−0.4275 (2)	0.0337 (7)	
H60A	0.136551	0.004820	−0.460269	0.040*	
H60B	0.169376	0.095403	−0.373601	0.040*	
C6C	0.0660 (2)	−0.3344 (2)	−0.0366 (2)	0.0316 (7)	
H81A	0.023323	−0.298391	−0.007095	0.038*	
H81B	0.068283	−0.330395	−0.089399	0.038*	
C3H	0.40532 (19)	0.4078 (2)	0.1539 (2)	0.0223 (5)	
H31	0.412370	0.350531	0.107019	0.027*	
C6O	0.8820 (2)	1.1276 (2)	1.2820 (3)	0.0355 (8)	
H15A	0.930640	1.094732	1.263202	0.043*	
H15B	0.890895	1.178657	1.271238	0.043*	
C1M	0.8143 (2)	1.3964 (2)	1.0673 (2)	0.0296 (6)	
H112	0.838949	1.446760	1.125538	0.035*	
C2A	0.3860 (2)	0.3583 (2)	0.3560 (2)	0.0261 (6)	
H23	0.382167	0.393452	0.417107	0.031*	
C1A	0.3026 (2)	0.3633 (2)	0.3001 (2)	0.0263 (6)	
H22	0.296500	0.426351	0.323651	0.032*	
C6M	0.9571 (2)	1.2972 (3)	0.9114 (2)	0.0360 (7)	
H16C	0.993496	1.275883	0.944716	0.043*	
H16D	0.949490	1.254439	0.850369	0.043*	
C1G	0.3322 (2)	0.2469 (2)	−0.2866 (2)	0.0261 (6)	
H36	0.325606	0.237890	−0.346046	0.031*	
C2G	0.4130 (2)	0.3225 (2)	−0.2295 (2)	0.0297 (6)	
H37	0.402220	0.377099	−0.231631	0.036*	
C5F	0.2709 (2)	0.0236 (2)	−0.4070 (2)	0.0313 (7)	
H43	0.259478	−0.000952	−0.367783	0.038*	
C5N	0.8291 (2)	1.3010 (2)	1.1942 (2)	0.0296 (6)	
H123	0.822373	1.237134	1.153070	0.035*	
C5A	0.2218 (2)	0.2201 (2)	0.2722 (3)	0.0354 (8)	
H26	0.228115	0.187738	0.211554	0.043*	0.5
H26A	0.242474	0.198121	0.216841	0.043*	0.5
C6P	0.8777 (2)	0.8298 (3)	1.0829 (3)	0.0488 (11)	
H15C	0.922669	0.815094	1.045935	0.059*	
H15D	0.875526	0.892869	1.103937	0.059*	
C1E	0.2557 (3)	−0.3821 (2)	−0.4396 (3)	0.0381 (8)	
H50	0.233598	−0.447115	−0.466634	0.046*	
C3F	0.4370 (2)	0.0555 (2)	−0.3430 (3)	0.0371 (8)	
H45	0.423245	0.037637	−0.299475	0.044*	
C4E	0.2992 (3)	−0.2274 (3)	−0.4629 (3)	0.0394 (8)	
H53	0.285868	−0.251608	−0.525977	0.047*	
C3E	0.3763 (3)	−0.2655 (3)	−0.4371 (3)	0.0381 (8)	
H52	0.394785	−0.233660	−0.373684	0.046*	
C2E	0.3468 (3)	−0.3648 (3)	−0.4666 (3)	0.0452 (9)	
H51	0.338054	−0.397941	−0.530001	0.054*	
O6H1	0.1267 (6)	0.4429 (7)	0.1842 (6)	0.069 (2)	0.5
H69A	0.084790	0.454334	0.158887	0.104*	0.5
O6H2	0.1390 (5)	0.4201 (6)	0.2070 (5)	0.0513 (18)	0.5
H69B	0.086549	0.414795	0.215377	0.077*	0.5
O6N	0.9921 (2)	1.3200 (4)	1.2466 (4)	0.0879 (15)	
H160	0.981500	1.326387	1.293386	0.132*	
O17W	0.0313 (3)	0.5385 (3)	0.0836 (3)	0.0830 (13)	
C6H	0.1430 (2)	0.3523 (3)	0.1262 (3)	0.0485 (10)	
H68A	0.096105	0.323025	0.073732	0.058*	0.5
H68B	0.140308	0.315691	0.155118	0.058*	0.5
H68C	0.097373	0.352977	0.082698	0.058*	0.5
H68D	0.128281	0.294788	0.125100	0.058*	0.5
C5E	0.2133 (3)	−0.2475 (3)	−0.4319 (4)	0.0491 (10)	
C2F	0.4473 (3)	−0.0271 (3)	−0.4240 (3)	0.0405 (8)	
H46	0.469776	−0.007000	−0.463401	0.049*	
C6D	0.0817 (3)	−0.3610 (3)	−0.3107 (3)	0.0457 (9)	
H85A	0.039178	−0.337689	−0.272430	0.055*	
H85B	0.096917	−0.320047	−0.333539	0.055*	
O6J2	0.8766 (4)	0.5577 (4)	0.5147 (4)	0.078 (2)	0.681 (10)
H96	0.839184	0.521215	0.518670	0.117*	0.681 (10)
C5J	0.7781 (3)	0.6352 (5)	0.5205 (5)	0.077 (2)	
H90	0.762506	0.694290	0.553033	0.093*	
C6N	0.9268 (3)	1.3497 (4)	1.2097 (4)	0.0577 (13)	
H15E	0.940345	1.342281	1.154716	0.069*	
H15F	0.931009	1.413403	1.247274	0.069*	
C6A2	0.1259 (9)	0.1885 (11)	0.2886 (9)	0.045 (3)	0.5
H74A	0.078314	0.194721	0.250531	0.053*	0.5
H74B	0.118894	0.125088	0.271841	0.053*	0.5
O6A2	0.1102 (5)	0.2371 (7)	0.3795 (6)	0.070 (2)	0.5
H76B	0.060 (8)	0.288 (8)	0.3835 (9)	0.105*	0.5
O6A1	0.0648 (3)	0.1665 (3)	0.1881 (4)	0.0340 (11)	0.5
H76A	0.049775	0.215614	0.206502	0.051*	0.5
C6A1	0.1262 (9)	0.1658 (9)	0.2527 (8)	0.034 (2)	0.5
H74C	0.130932	0.103981	0.236015	0.041*	0.5
H74D	0.101785	0.188688	0.305482	0.041*	0.5
C6J	0.8707 (4)	0.6430 (4)	0.5734 (5)	0.0787 (16)	
H95A	0.918469	0.689071	0.577207	0.094*	0.681 (10)
H95B	0.867133	0.650010	0.630629	0.094*	0.681 (10)
H95C	0.889915	0.703318	0.621860	0.094*	0.319 (10)
H95D	0.864555	0.601232	0.596598	0.094*	0.319 (10)
O6E1	0.0955 (12)	−0.2315 (11)	−0.5249 (10)	0.135 (5)	0.5
H3A	0.120128	−0.271701	−0.555845	0.203*	0.5
C6E1	0.1300 (10)	−0.2042 (10)	−0.4412 (11)	0.059 (3)	0.5
H58A	0.082986	−0.220544	−0.414892	0.071*	0.5
H58B	0.148970	−0.139331	−0.410665	0.071*	0.5
O6E2	0.0580 (4)	−0.2339 (6)	−0.4395 (8)	0.082 (3)	0.5
H3B	0.023202	−0.203576	−0.447411	0.123*	0.5
C6E2	0.1375 (9)	−0.2283 (10)	−0.4785 (10)	0.056 (3)	0.5
H58C	0.153946	−0.168810	−0.472727	0.068*	0.5
H58D	0.124115	−0.271888	−0.539680	0.068*	0.5
O18W	0.7415 (3)	0.6151 (3)	0.1973 (3)	0.0746 (11)	
O3W	0.57812 (19)	0.6781 (2)	0.2082 (2)	0.0427 (6)	
H2WA	0.543447	0.627810	0.192091	0.064*	
H2WB	0.562760	0.691511	0.168178	0.064*	
O5W	0.95468 (17)	0.42708 (19)	0.65887 (19)	0.0413 (6)	
H3WA	0.982321	0.386821	0.660296	0.062*	
H3WB	0.963261	0.467232	0.711564	0.062*	
O4W	0.8557 (2)	0.3241 (2)	0.4879 (2)	0.0524 (7)	
H4WA	0.853898	0.290389	0.511504	0.079*	
H4WB	0.824511	0.363095	0.514489	0.079*	
O6W	0.66308 (17)	0.27742 (19)	0.45749 (18)	0.0377 (5)	
H5WA	0.613768	0.289410	0.440276	0.057*	
H5WB	0.650579	0.235219	0.470067	0.057*	
O9W	0.96086 (17)	1.03482 (18)	0.41120 (18)	0.0377 (5)	
H6WA	0.917160	0.998846	0.412368	0.056*	
H6WB	0.975257	1.077754	0.462820	0.056*	
O7W	0.07383 (16)	0.32250 (18)	0.67389 (19)	0.0366 (5)	
H7WA	0.120021	0.308498	0.650747	0.055*	
H7WB	0.086153	0.321928	0.721890	0.055*	
O8W	0.18028 (19)	0.27077 (19)	0.5486 (2)	0.0429 (6)	
H8WA	0.177603	0.218572	0.540680	0.064*	
H8WB	0.143470	0.295326	0.530023	0.064*	
O19W	0.9146 (4)	0.6597 (4)	0.1674 (4)	0.1066 (18)	
O15W	0.0418 (7)	0.7128 (9)	0.3110 (8)	0.192 (4)	
O14W	0.9950 (5)	0.3989 (7)	0.4198 (5)	0.155 (4)	
O6K1	0.9611 (8)	0.8782 (8)	0.499 (2)	0.084 (6)	0.53 (4)
H1D	0.971005	0.872130	0.542193	0.126*	0.53 (4)
C6K1	0.8809 (11)	0.8167 (16)	0.4420 (16)	0.044 (4)	0.53 (4)
H6A	0.871216	0.765747	0.452968	0.053*	0.53 (4)
H6B	0.888546	0.794613	0.382403	0.053*	0.53 (4)
O10W	0.1552 (6)	0.3800 (7)	0.4684 (6)	0.133 (2)	
H0AA	0.203617	0.378294	0.447614	0.199*	
H0AB	0.123652	0.406403	0.447476	0.199*	
O13W	0.9767 (9)	0.5847 (7)	0.4050 (8)	0.118 (3)	0.578 (12)
O6J1	0.9400 (6)	0.6234 (8)	0.5190 (8)	0.069 (3)	0.319 (10)
H96A	0.914887	0.590926	0.468113	0.103*	0.319 (10)
O16W	0.1722 (5)	0.6001 (6)	0.2501 (7)	0.153 (3)	
O12W	0.9259 (11)	0.5828 (9)	0.3209 (11)	0.111 (4)	0.422 (12)
O6K2	0.9625 (8)	0.8655 (11)	0.448 (2)	0.068 (5)	0.47 (4)
H1E	0.999543	0.838023	0.457628	0.103*	0.47 (4)
C6K2	0.8742 (13)	0.8103 (18)	0.4156 (19)	0.046 (5)	0.47 (4)
H6AA	0.874542	0.760504	0.428168	0.055*	0.47 (4)
H6AB	0.859853	0.785497	0.353011	0.055*	0.47 (4)

### Atomic displacement parameters (Å^2^)

**Table:**

	*U*^11^	*U*^22^	*U*^33^	*U*^12^	*U*^13^	*U*^23^
Li1	0.027 (2)	0.031 (2)	0.021 (2)	0.0053 (19)	0.0061 (19)	0.010 (2)
Li2	0.047 (3)	0.050 (4)	0.048 (4)	0.008 (3)	0.010 (3)	0.030 (3)
O4G	0.0276 (10)	0.0275 (10)	0.0177 (10)	0.0093 (8)	0.0072 (8)	0.0131 (8)
O4B	0.0257 (9)	0.0217 (9)	0.0178 (10)	0.0045 (7)	0.0044 (8)	0.0059 (8)
O4I	0.0237 (9)	0.0258 (10)	0.0237 (11)	0.0091 (8)	0.0037 (8)	0.0096 (9)
O5L	0.0189 (9)	0.0267 (10)	0.0251 (11)	0.0024 (8)	0.0056 (8)	0.0071 (9)
O4M	0.0244 (9)	0.0237 (9)	0.0216 (11)	0.0054 (7)	0.0071 (8)	0.0100 (8)
O5D	0.0190 (9)	0.0377 (11)	0.0243 (11)	0.0022 (8)	0.0015 (8)	0.0154 (10)
O4C	0.0259 (9)	0.0253 (10)	0.0178 (10)	0.0007 (8)	0.0034 (8)	0.0088 (8)
O4L	0.0304 (10)	0.0182 (9)	0.0206 (10)	0.0023 (8)	0.0015 (8)	0.0063 (8)
O2K	0.0219 (9)	0.0248 (10)	0.0288 (12)	0.0062 (8)	0.0013 (8)	0.0099 (9)
O5B	0.0202 (9)	0.0202 (9)	0.0233 (11)	0.0017 (7)	0.0069 (8)	0.0044 (8)
O4P	0.0235 (9)	0.0216 (9)	0.0207 (10)	0.0045 (7)	0.0059 (8)	0.0076 (8)
O2P	0.0267 (10)	0.0263 (10)	0.0269 (12)	−0.0030 (8)	0.0000 (9)	0.0085 (9)
O3B	0.0167 (8)	0.0245 (9)	0.0287 (11)	0.0074 (7)	0.0043 (8)	0.0103 (9)
O3C	0.0178 (9)	0.0283 (10)	0.0245 (11)	0.0060 (8)	0.0042 (8)	0.0083 (9)
O5C	0.0183 (9)	0.0311 (10)	0.0212 (11)	0.0001 (8)	0.0022 (8)	0.0060 (9)
O4H	0.0314 (11)	0.0278 (10)	0.0231 (11)	0.0036 (8)	0.0080 (9)	0.0136 (9)
O4F	0.0372 (12)	0.0312 (11)	0.0226 (11)	0.0115 (9)	0.0081 (9)	0.0112 (9)
O4A	0.0256 (10)	0.0253 (10)	0.0231 (11)	0.0071 (8)	0.0049 (8)	0.0114 (9)
O3L	0.0258 (10)	0.0244 (10)	0.0265 (11)	0.0061 (8)	0.0006 (8)	0.0103 (9)
O5O	0.0215 (9)	0.0235 (10)	0.0266 (11)	0.0023 (8)	0.0007 (8)	0.0104 (9)
O5K	0.0233 (10)	0.0243 (10)	0.0433 (14)	0.0067 (8)	0.0109 (9)	0.0164 (10)
O4J	0.0352 (11)	0.0259 (10)	0.0312 (12)	0.0064 (9)	0.0006 (9)	0.0165 (10)
O3A	0.0231 (9)	0.0288 (10)	0.0231 (11)	0.0022 (8)	0.0014 (8)	0.0123 (9)
O3H	0.0196 (9)	0.0317 (11)	0.0229 (11)	0.0022 (8)	0.0008 (8)	0.0149 (9)
O3O	0.0229 (10)	0.0270 (10)	0.0418 (14)	0.0082 (8)	0.0128 (9)	0.0179 (10)
O2O	0.0218 (9)	0.0236 (10)	0.0344 (12)	0.0027 (8)	0.0102 (9)	0.0083 (9)
O3G	0.0246 (10)	0.0279 (10)	0.0266 (12)	0.0021 (8)	0.0039 (8)	0.0107 (9)
O4K	0.0438 (12)	0.0252 (10)	0.0231 (11)	0.0028 (9)	0.0000 (9)	0.0144 (9)
O5H	0.0260 (10)	0.0474 (13)	0.0307 (13)	0.0143 (9)	0.0088 (9)	0.0219 (11)
O3D	0.0288 (10)	0.0297 (10)	0.0258 (11)	0.0128 (8)	0.0109 (9)	0.0130 (9)
O2D	0.0368 (11)	0.0317 (11)	0.0211 (11)	0.0158 (9)	0.0057 (9)	0.0117 (9)
O5P	0.0214 (9)	0.0288 (11)	0.0265 (12)	0.0092 (8)	−0.0007 (8)	0.0005 (9)
O4O	0.0293 (10)	0.0239 (10)	0.0215 (11)	0.0039 (8)	0.0063 (8)	0.0112 (9)
O3M	0.0325 (11)	0.0424 (12)	0.0288 (12)	0.0187 (10)	0.0094 (9)	0.0188 (11)
O6G	0.0284 (11)	0.0391 (12)	0.0377 (14)	0.0136 (9)	0.0111 (10)	0.0203 (11)
O5I	0.0294 (10)	0.0322 (11)	0.0254 (12)	0.0099 (9)	0.0045 (9)	0.0147 (9)
O3P	0.0241 (10)	0.0247 (10)	0.0304 (12)	0.0017 (8)	0.0097 (9)	0.0088 (9)
O2B	0.0162 (9)	0.0263 (10)	0.0272 (12)	−0.0003 (7)	0.0027 (8)	0.0099 (9)
O4N	0.0281 (10)	0.0303 (10)	0.0259 (12)	0.0031 (8)	0.0097 (9)	0.0158 (9)
O4D	0.0337 (11)	0.0290 (10)	0.0250 (11)	0.0104 (9)	0.0085 (9)	0.0153 (9)
O2L	0.0337 (11)	0.0314 (11)	0.0272 (12)	0.0167 (9)	0.0069 (9)	0.0113 (10)
O5N	0.0312 (11)	0.0329 (11)	0.0300 (12)	−0.0047 (9)	0.0000 (9)	0.0174 (10)
O5A	0.0254 (10)	0.0377 (12)	0.0467 (15)	0.0103 (9)	0.0143 (10)	0.0294 (11)
O2C	0.0264 (10)	0.0281 (10)	0.0248 (11)	0.0073 (8)	−0.0019 (9)	0.0102 (9)
O1W	0.0307 (11)	0.0380 (12)	0.0268 (12)	0.0106 (9)	0.0079 (9)	0.0179 (10)
O5M	0.0220 (10)	0.0572 (15)	0.0266 (12)	0.0026 (10)	0.0051 (9)	0.0251 (12)
O2H	0.0272 (10)	0.0404 (12)	0.0313 (12)	0.0026 (9)	0.0070 (9)	0.0214 (11)
O5G	0.0244 (10)	0.0325 (11)	0.0232 (11)	0.0055 (8)	0.0023 (8)	0.0115 (9)
O6I	0.0284 (11)	0.0371 (12)	0.0310 (13)	0.0056 (9)	−0.0047 (9)	0.0147 (10)
O4E	0.0482 (14)	0.0338 (12)	0.0355 (14)	0.0131 (10)	0.0187 (11)	0.0202 (11)
O2I	0.0382 (12)	0.0344 (12)	0.0311 (13)	−0.0112 (10)	−0.0083 (10)	0.0187 (11)
O3J	0.0252 (10)	0.0351 (12)	0.0308 (13)	0.0034 (9)	0.0059 (9)	0.0146 (10)
O5F	0.0595 (16)	0.0269 (11)	0.0254 (12)	0.0127 (11)	0.0098 (11)	0.0107 (10)
O2A	0.0278 (10)	0.0289 (10)	0.0275 (12)	−0.0039 (8)	−0.0004 (9)	0.0143 (9)
O2N	0.0320 (11)	0.0374 (12)	0.0510 (16)	0.0139 (10)	0.0243 (11)	0.0251 (12)
O6L	0.0302 (12)	0.0419 (13)	0.0400 (15)	0.0072 (10)	0.0163 (11)	0.0051 (12)
O6C	0.0347 (12)	0.0447 (14)	0.0371 (15)	−0.0055 (10)	−0.0064 (11)	0.0186 (12)
O3K	0.0249 (10)	0.0327 (11)	0.0466 (15)	0.0011 (9)	0.0030 (10)	0.0227 (11)
O3I	0.0191 (10)	0.0617 (16)	0.0351 (14)	−0.0032 (10)	0.0002 (9)	0.0288 (13)
O6F	0.0508 (14)	0.0344 (12)	0.0265 (13)	0.0116 (10)	0.0097 (11)	0.0134 (10)
O2G	0.0317 (12)	0.0638 (17)	0.0254 (13)	−0.0032 (12)	0.0122 (10)	0.0087 (12)
O11W	0.0449 (14)	0.0532 (16)	0.0398 (16)	0.0007 (12)	0.0043 (12)	0.0266 (14)
O2W	0.0308 (12)	0.0347 (12)	0.0514 (17)	0.0079 (10)	0.0173 (11)	0.0083 (12)
O2J	0.0403 (13)	0.0380 (13)	0.0302 (13)	0.0109 (10)	−0.0016 (10)	0.0122 (11)
O6O	0.0394 (13)	0.0330 (12)	0.0424 (16)	0.0026 (10)	−0.0088 (11)	0.0077 (12)
O6M	0.0319 (12)	0.0534 (15)	0.0427 (16)	0.0057 (11)	0.0129 (11)	0.0185 (13)
O3N	0.0389 (13)	0.0607 (16)	0.0488 (17)	0.0254 (12)	0.0172 (12)	0.0388 (15)
O2M	0.0459 (13)	0.0434 (13)	0.0327 (13)	0.0204 (11)	0.0204 (11)	0.0202 (11)
O6B	0.0262 (11)	0.0458 (14)	0.0391 (15)	0.0083 (10)	0.0063 (10)	0.0051 (12)
O3F	0.0313 (12)	0.0432 (14)	0.066 (2)	0.0104 (11)	0.0096 (13)	0.0230 (14)
O5J	0.0390 (15)	0.099 (3)	0.127 (3)	0.0363 (16)	0.0404 (18)	0.101 (3)
O3E	0.0547 (16)	0.0431 (14)	0.0536 (18)	0.0180 (12)	0.0290 (14)	0.0278 (14)
O5E	0.0576 (17)	0.0429 (15)	0.0506 (18)	−0.0049 (12)	−0.0137 (14)	0.0316 (14)
O2E	0.076 (2)	0.0560 (17)	0.073 (2)	0.0421 (16)	0.0515 (18)	0.0479 (17)
O6D	0.0362 (14)	0.097 (3)	0.058 (2)	0.0103 (15)	−0.0048 (13)	0.053 (2)
O2F	0.0399 (14)	0.0421 (15)	0.068 (2)	0.0170 (12)	0.0177 (14)	0.0172 (14)
O6P	0.0441 (16)	0.0523 (18)	0.066 (2)	0.0100 (13)	−0.0227 (16)	0.0009 (16)
C3O	0.0193 (12)	0.0242 (13)	0.0229 (14)	0.0056 (10)	0.0060 (10)	0.0090 (11)
C2L	0.0263 (13)	0.0223 (13)	0.0218 (14)	0.0065 (11)	0.0074 (11)	0.0107 (12)
C1K	0.0208 (12)	0.0227 (13)	0.0244 (15)	0.0063 (10)	0.0059 (11)	0.0106 (11)
C4B	0.0204 (12)	0.0242 (13)	0.0196 (14)	0.0041 (10)	0.0037 (10)	0.0068 (11)
C1L	0.0225 (13)	0.0249 (13)	0.0246 (15)	0.0058 (10)	0.0077 (11)	0.0112 (12)
C1B	0.0208 (12)	0.0222 (13)	0.0217 (14)	0.0033 (10)	0.0032 (10)	0.0095 (11)
C5I	0.0245 (13)	0.0232 (13)	0.0266 (16)	0.0049 (10)	0.0043 (12)	0.0091 (12)
C1H	0.0293 (14)	0.0265 (13)	0.0219 (15)	0.0081 (11)	0.0083 (12)	0.0122 (12)
C2K	0.0199 (12)	0.0226 (12)	0.0213 (14)	0.0052 (10)	0.0043 (10)	0.0100 (11)
C3M	0.0228 (13)	0.0280 (14)	0.0236 (15)	0.0057 (11)	0.0061 (11)	0.0126 (12)
C4K	0.0256 (13)	0.0251 (13)	0.0219 (15)	0.0026 (11)	0.0036 (11)	0.0127 (12)
C4L	0.0205 (12)	0.0192 (12)	0.0241 (14)	0.0035 (10)	0.0028 (11)	0.0094 (11)
C4C	0.0208 (12)	0.0246 (13)	0.0210 (14)	0.0036 (10)	0.0019 (11)	0.0100 (11)
C1I	0.0367 (15)	0.0236 (13)	0.0237 (15)	0.0069 (11)	0.0044 (12)	0.0137 (12)
C5L	0.0199 (13)	0.0264 (14)	0.0253 (15)	0.0057 (10)	0.0033 (11)	0.0080 (12)
C3L	0.0193 (12)	0.0256 (13)	0.0227 (14)	0.0043 (10)	0.0016 (11)	0.0119 (12)
C1D	0.0244 (13)	0.0233 (13)	0.0183 (14)	0.0011 (10)	0.0005 (11)	0.0071 (11)
C5B	0.0171 (12)	0.0230 (13)	0.0216 (14)	0.0030 (10)	0.0034 (10)	0.0031 (11)
C4O	0.0256 (13)	0.0242 (13)	0.0223 (15)	0.0036 (11)	0.0049 (11)	0.0128 (12)
C3B	0.0175 (12)	0.0239 (13)	0.0227 (14)	0.0068 (10)	0.0057 (10)	0.0099 (11)
C3K	0.0244 (13)	0.0277 (14)	0.0280 (16)	0.0042 (11)	0.0086 (12)	0.0162 (13)
C4I	0.0196 (12)	0.0219 (12)	0.0199 (14)	0.0008 (10)	0.0018 (11)	0.0050 (11)
C4H	0.0250 (13)	0.0318 (15)	0.0229 (15)	0.0052 (11)	0.0064 (11)	0.0150 (13)
C5O	0.0251 (14)	0.0255 (13)	0.0296 (16)	0.0024 (11)	0.0060 (12)	0.0141 (13)
C4P	0.0193 (12)	0.0225 (13)	0.0221 (15)	0.0036 (10)	0.0023 (11)	0.0047 (11)
C5C	0.0240 (13)	0.0308 (15)	0.0209 (15)	0.0063 (11)	0.0043 (11)	0.0085 (12)
C2D	0.0272 (13)	0.0222 (13)	0.0192 (14)	0.0039 (10)	0.0008 (11)	0.0079 (11)
C1P	0.0235 (13)	0.0257 (13)	0.0205 (14)	0.0059 (11)	−0.0015 (11)	0.0074 (12)
C2P	0.0246 (13)	0.0237 (13)	0.0222 (15)	0.0038 (11)	0.0014 (11)	0.0084 (12)
C4N	0.0262 (14)	0.0295 (14)	0.0279 (16)	0.0059 (11)	0.0106 (12)	0.0172 (13)
C3C	0.0186 (12)	0.0226 (13)	0.0238 (15)	0.0039 (10)	0.0024 (11)	0.0094 (11)
C2C	0.0228 (12)	0.0231 (13)	0.0197 (14)	0.0051 (10)	0.0016 (11)	0.0077 (11)
C3P	0.0218 (12)	0.0227 (12)	0.0200 (14)	0.0043 (10)	0.0047 (11)	0.0068 (11)
C4D	0.0269 (13)	0.0266 (13)	0.0236 (15)	0.0071 (11)	0.0068 (11)	0.0128 (12)
C3A	0.0223 (13)	0.0257 (13)	0.0209 (14)	0.0031 (10)	0.0039 (11)	0.0107 (11)
C2H	0.0311 (14)	0.0243 (13)	0.0225 (15)	0.0056 (11)	0.0066 (12)	0.0102 (12)
C5P	0.0204 (13)	0.0267 (14)	0.0286 (17)	0.0022 (11)	0.0044 (12)	0.0006 (13)
C3G	0.0229 (13)	0.0250 (13)	0.0206 (14)	0.0006 (10)	0.0040 (11)	0.0103 (12)
C4M	0.0188 (12)	0.0285 (14)	0.0221 (15)	0.0045 (10)	0.0042 (10)	0.0121 (12)
C4G	0.0234 (13)	0.0265 (13)	0.0169 (14)	0.0056 (10)	0.0045 (10)	0.0108 (11)
C6G	0.0226 (13)	0.0368 (16)	0.0340 (18)	0.0029 (12)	0.0034 (12)	0.0193 (14)
C2O	0.0211 (12)	0.0262 (13)	0.0249 (15)	0.0040 (10)	0.0080 (11)	0.0118 (12)
C1C	0.0203 (12)	0.0250 (13)	0.0179 (14)	0.0015 (10)	0.0024 (11)	0.0051 (11)
C3D	0.0247 (13)	0.0234 (13)	0.0222 (14)	0.0077 (10)	0.0068 (11)	0.0093 (11)
C3I	0.0234 (13)	0.0350 (15)	0.0249 (16)	−0.0007 (11)	−0.0008 (12)	0.0142 (13)
C2M	0.0354 (16)	0.0277 (14)	0.0281 (17)	0.0071 (12)	0.0124 (13)	0.0144 (13)
C1N	0.0337 (15)	0.0228 (13)	0.0240 (15)	0.0023 (11)	0.0110 (12)	0.0120 (12)
C5M	0.0245 (14)	0.0502 (19)	0.0247 (16)	0.0143 (13)	0.0090 (12)	0.0225 (15)
C6I	0.0218 (13)	0.0327 (16)	0.0299 (17)	0.0009 (11)	0.0036 (12)	0.0097 (14)
C2B	0.0165 (12)	0.0223 (13)	0.0262 (15)	0.0007 (10)	0.0040 (10)	0.0118 (12)
C1O	0.0222 (13)	0.0245 (13)	0.0206 (14)	0.0027 (10)	0.0051 (11)	0.0093 (11)
C3N	0.0230 (13)	0.0398 (16)	0.0333 (18)	0.0109 (12)	0.0113 (12)	0.0231 (15)
C4J	0.0294 (15)	0.0377 (16)	0.0410 (19)	0.0104 (13)	0.0108 (14)	0.0295 (16)
C3J	0.0221 (13)	0.0261 (13)	0.0282 (16)	0.0053 (10)	0.0022 (11)	0.0128 (12)
C4A	0.0226 (13)	0.0289 (14)	0.0319 (17)	0.0053 (11)	0.0091 (12)	0.0193 (13)
C2N	0.0318 (15)	0.0290 (14)	0.0353 (18)	0.0089 (12)	0.0183 (13)	0.0170 (14)
C2J	0.0373 (16)	0.0269 (14)	0.0291 (17)	0.0068 (12)	0.0053 (13)	0.0129 (13)
C4F	0.0377 (16)	0.0293 (15)	0.0269 (17)	0.0085 (13)	0.0123 (13)	0.0119 (13)
C2I	0.0323 (15)	0.0253 (13)	0.0204 (15)	−0.0014 (11)	−0.0014 (12)	0.0100 (12)
C5H	0.0208 (13)	0.0477 (18)	0.0288 (17)	0.0035 (12)	0.0034 (12)	0.0212 (15)
C5G	0.0251 (13)	0.0264 (13)	0.0242 (15)	0.0032 (11)	0.0019 (11)	0.0123 (12)
C6B	0.0203 (13)	0.0274 (15)	0.046 (2)	−0.0004 (11)	0.0104 (13)	0.0056 (14)
C1J	0.0401 (17)	0.0327 (16)	0.0361 (19)	0.0137 (13)	0.0119 (14)	0.0228 (15)
C5D	0.0261 (14)	0.0391 (16)	0.0342 (18)	0.0139 (12)	0.0125 (13)	0.0218 (15)
C6L	0.0245 (14)	0.0336 (16)	0.0316 (18)	0.0096 (12)	−0.0009 (12)	0.0052 (14)
C1F	0.056 (2)	0.0309 (16)	0.0316 (18)	0.0152 (15)	0.0199 (16)	0.0168 (15)
C5K	0.0233 (14)	0.0201 (13)	0.045 (2)	0.0018 (11)	−0.0001 (13)	0.0140 (13)
C6F	0.0347 (16)	0.0329 (16)	0.0268 (17)	0.0011 (13)	0.0051 (13)	0.0102 (14)
C6C	0.0230 (14)	0.0370 (16)	0.0280 (17)	0.0056 (12)	0.0017 (12)	0.0106 (14)
C3H	0.0205 (12)	0.0274 (13)	0.0184 (14)	0.0038 (10)	0.0026 (10)	0.0109 (11)
C6O	0.0226 (14)	0.0317 (16)	0.049 (2)	0.0009 (12)	0.0009 (14)	0.0193 (16)
C1M	0.0318 (15)	0.0349 (15)	0.0234 (16)	0.0006 (12)	0.0085 (12)	0.0156 (13)
C2A	0.0280 (14)	0.0279 (14)	0.0256 (16)	0.0037 (11)	0.0067 (12)	0.0156 (13)
C1A	0.0284 (14)	0.0300 (14)	0.0278 (16)	0.0076 (11)	0.0119 (12)	0.0180 (13)
C6M	0.0240 (14)	0.054 (2)	0.0304 (18)	0.0125 (13)	0.0050 (13)	0.0191 (16)
C1G	0.0296 (14)	0.0283 (14)	0.0200 (14)	0.0042 (11)	0.0052 (11)	0.0113 (12)
C2G	0.0304 (15)	0.0356 (16)	0.0199 (15)	0.0018 (12)	0.0076 (12)	0.0109 (13)
C5F	0.0406 (17)	0.0267 (14)	0.0251 (16)	0.0061 (12)	0.0108 (13)	0.0102 (13)
C5N	0.0226 (14)	0.0404 (17)	0.0346 (18)	0.0053 (12)	0.0077 (12)	0.0250 (15)
C5A	0.0206 (14)	0.0377 (17)	0.055 (2)	0.0019 (12)	0.0032 (14)	0.0300 (17)
C6P	0.0210 (15)	0.0384 (19)	0.053 (3)	−0.0012 (13)	0.0068 (15)	−0.0049 (18)
C1E	0.055 (2)	0.0325 (16)	0.0299 (18)	0.0085 (15)	0.0056 (15)	0.0180 (15)
C3F	0.0360 (17)	0.0363 (17)	0.041 (2)	0.0100 (14)	0.0170 (15)	0.0168 (16)
C4E	0.058 (2)	0.0360 (17)	0.0280 (18)	0.0088 (15)	0.0089 (16)	0.0181 (15)
C3E	0.049 (2)	0.0379 (18)	0.038 (2)	0.0171 (15)	0.0206 (16)	0.0217 (16)
C2E	0.069 (3)	0.0356 (18)	0.039 (2)	0.0175 (17)	0.0220 (19)	0.0202 (17)
O6H1	0.041 (4)	0.109 (7)	0.052 (5)	0.021 (4)	0.022 (4)	0.028 (4)
O6H2	0.033 (3)	0.096 (5)	0.051 (4)	0.017 (3)	0.014 (3)	0.055 (4)
O6N	0.0421 (18)	0.140 (4)	0.137 (4)	0.028 (2)	0.023 (2)	0.109 (4)
O17W	0.072	0.072 (2)	0.074 (3)	−0.0193 (19)	0.027 (2)	0.013 (2)
C6H	0.0212 (15)	0.081 (3)	0.049 (2)	0.0025 (16)	0.0027 (15)	0.038 (2)
C5E	0.044 (2)	0.0411 (19)	0.076 (3)	0.0085 (16)	0.004 (2)	0.041 (2)
C2F	0.047 (2)	0.0387 (18)	0.046 (2)	0.0185 (15)	0.0291 (17)	0.0215 (17)
C6D	0.0302 (17)	0.074 (3)	0.059 (3)	0.0261 (17)	0.0153 (17)	0.047 (2)
O6J2	0.069 (3)	0.070 (3)	0.074 (4)	−0.016 (3)	0.024 (3)	0.020 (3)
C5J	0.0209 (16)	0.137 (5)	0.137 (5)	−0.003 (2)	−0.002 (2)	0.127 (5)
C6N	0.0256 (17)	0.093 (4)	0.084 (4)	0.0039 (19)	0.0072 (19)	0.069 (3)
C6A2	0.020 (3)	0.057 (8)	0.072 (7)	0.001 (4)	0.000 (6)	0.046 (7)
O6A2	0.047 (3)	0.108 (6)	0.089 (5)	0.026 (4)	0.026 (4)	0.069 (5)
O6A1	0.0156 (18)	0.038 (2)	0.046 (3)	0.0006 (16)	−0.0087 (18)	0.023 (2)
C6A1	0.022 (3)	0.039 (6)	0.049 (6)	0.003 (3)	0.003 (5)	0.030 (5)
C6J	0.065 (3)	0.089 (4)	0.093 (5)	0.012 (3)	0.008 (3)	0.055 (4)
O6E1	0.149 (9)	0.142 (9)	0.112 (7)	0.066 (7)	0.000 (7)	0.056 (7)
C6E1	0.046 (5)	0.052 (7)	0.092 (8)	0.008 (4)	0.008 (6)	0.046 (7)
O6E2	0.017 (2)	0.080 (4)	0.165 (8)	0.005 (3)	0.005 (3)	0.075 (5)
C6E2	0.046 (5)	0.054 (7)	0.073 (8)	0.007 (4)	−0.010 (5)	0.040 (6)
O18W	0.083 (3)	0.070 (2)	0.056 (2)	0.028 (2)	0.013 (2)	0.015 (2)
O3W	0.0425 (14)	0.0472 (15)	0.0399 (15)	0.0069 (11)	0.0045 (11)	0.0233 (13)
O5W	0.0321 (12)	0.0436 (14)	0.0367 (15)	0.0150 (10)	0.0006 (11)	0.0091 (12)
O4W	0.0497 (16)	0.0530 (17)	0.0395 (17)	0.0101 (13)	0.0007 (13)	0.0112 (14)
O6W	0.0341 (12)	0.0453 (14)	0.0363 (14)	0.0068 (10)	0.0077 (10)	0.0216 (12)
O9W	0.0324 (12)	0.0399 (13)	0.0335 (14)	0.0025 (10)	0.0077 (10)	0.0122 (11)
O7W	0.0292 (11)	0.0438 (13)	0.0378 (14)	0.0112 (10)	0.0057 (10)	0.0196 (12)
O8W	0.0405 (13)	0.0429 (14)	0.0531 (18)	0.0060 (11)	0.0137 (12)	0.0288 (14)
O19W	0.091 (3)	0.115 (4)	0.099 (4)	0.033 (3)	0.005 (3)	0.038 (3)
O15W	0.141 (6)	0.253 (10)	0.153 (7)	0.067 (7)	−0.007 (5)	0.074 (7)
O14W	0.108 (5)	0.212 (9)	0.112 (5)	−0.052 (5)	−0.026 (4)	0.080 (6)
O6K1	0.037 (4)	0.038 (4)	0.137 (14)	0.005 (3)	−0.019 (7)	0.017 (7)
C6K1	0.017 (4)	0.034 (5)	0.078 (11)	0.009 (3)	0.000 (5)	0.025 (7)
O10W	0.132 (6)	0.155 (7)	0.134 (7)	0.057 (5)	0.042 (5)	0.076 (5)
O13W	0.139 (7)	0.085 (5)	0.112 (7)	0.030 (5)	0.060 (6)	0.023 (5)
O6J1	0.020 (4)	0.074 (6)	0.116 (8)	−0.001 (4)	0.003 (4)	0.053 (6)
O16W	0.099 (4)	0.140 (5)	0.221 (8)	0.015 (4)	0.064 (5)	0.081 (5)
O12W	0.128 (7)	0.083 (5)	0.117 (7)	0.024 (5)	0.067 (6)	0.031 (5)
O6K2	0.024 (3)	0.038 (5)	0.124 (13)	0.004 (3)	−0.004 (6)	0.028 (7)
C6K2	0.025 (5)	0.031 (5)	0.078 (12)	0.008 (4)	0.001 (5)	0.024 (8)

### Geometric parameters (Å, º)

**Table:**

Li1—O2W	1.882 (6)	C5B—H19	0.9800
Li1—O1W	1.908 (6)	C4O—C5O	1.521 (4)
Li1—O2I	1.921 (6)	C4O—H129	0.9800
Li1—O3A	1.977 (6)	C3B—C2B	1.501 (4)
Li1—H17B	2.3333	C3B—H17	0.9800
Li2—O2J	1.902 (8)	C3K—H100	0.9800
Li2—O11W	1.954 (8)	C4I—C3I	1.518 (4)
Li2—O3E^i^	1.973 (8)	C4I—H141	0.9800
Li2—O3K	1.979 (7)	C4H—C5H	1.520 (4)
O4G—C1H	1.413 (4)	C4H—C3H	1.524 (4)
O4G—C4G	1.445 (4)	C4H—H32	0.9800
O4B—C1C	1.407 (4)	C5O—C6O	1.516 (4)
O4B—C4B	1.429 (4)	C5O—H130	0.9800
O4I—C1P	1.412 (4)	C4P—C5P	1.521 (4)
O4I—C4I	1.435 (4)	C4P—C3P	1.521 (4)
O5L—C1L	1.412 (3)	C4P—H136	0.9800
O5L—C5L	1.428 (4)	C5C—C6C	1.497 (4)
O4M—C1L	1.415 (4)	C5C—H12	0.9800
O4M—C4M	1.437 (4)	C2D—C3D	1.528 (4)
O5D—C1D	1.407 (4)	C2D—H2	0.9800
O5D—C5D	1.439 (4)	C1P—C2P	1.522 (4)
O4C—C1D	1.419 (4)	C1P—H133	0.9800
O4C—C4C	1.436 (4)	C2P—C3P	1.513 (4)
O4L—C1K	1.401 (4)	C2P—H134	0.9800
O4L—C4L	1.447 (3)	C4N—C5N	1.527 (4)
O2K—C2K	1.421 (3)	C4N—C3N	1.528 (4)
O2K—H171	0.8200	C4N—H122	0.9800
O5B—C1B	1.420 (3)	C3C—C2C	1.513 (4)
O5B—C5B	1.442 (3)	C3C—H10	0.9800
O4P—C1O	1.409 (4)	C2C—C1C	1.540 (4)
O4P—C4P	1.439 (3)	C2C—H9	0.9800
O2P—C2P	1.430 (4)	C3P—H135	0.9800
O2P—H154	0.8200	C4D—C3D	1.520 (4)
O3B—C3B	1.415 (3)	C4D—C5D	1.524 (4)
O3B—H79	0.8200	C4D—H4	0.9800
O3C—C3C	1.419 (3)	C3A—C2A	1.512 (4)
O3C—H83	0.8200	C3A—C4A	1.519 (4)
O5C—C1C	1.404 (3)	C3A—H24	0.9800
O5C—C5C	1.448 (4)	C2H—C3H	1.510 (4)
O4H—C1A	1.417 (4)	C2H—H30	0.9800
O4H—C4H	1.439 (4)	C5P—C6P	1.513 (4)
O4F—C4F	1.414 (4)	C5P—H137	0.9800
O4F—C1G	1.428 (4)	C3G—C4G	1.517 (4)
O4A—C1B	1.409 (4)	C3G—C2G	1.527 (4)
O4A—C4A	1.439 (4)	C3G—H38	0.9800
O3L—C3L	1.437 (4)	C4M—C5M	1.524 (4)
O3L—H170	0.8200	C4M—H115	0.9800
O5O—C1O	1.408 (3)	C4G—C5G	1.523 (4)
O5O—C5O	1.443 (4)	C4G—H39	0.9800
O5K—C1K	1.417 (3)	C6G—C5G	1.509 (4)
O5K—C5K	1.448 (4)	C6G—H64A	0.9700
O4J—C1I	1.420 (4)	C6G—H64B	0.9700
O4J—C4J	1.420 (4)	C2O—C1O	1.539 (4)
O3A—C3A	1.442 (4)	C2O—H127	0.9800
O3H—C3H	1.423 (3)	C1C—H8	0.9800
O3H—H70	0.8200	C3D—H3	0.9800
O3O—C3O	1.437 (3)	C3I—C2I	1.525 (5)
O3O—H157	0.8200	C3I—H142	0.9800
O2O—C2O	1.401 (3)	C2M—C1M	1.521 (5)
O2O—H158	0.8200	C2M—H113	0.9800
O3G—C3G	1.425 (3)	C1N—C2N	1.534 (5)
O3G—H67	0.8200	C1N—H119	0.9800
O4K—C1J	1.410 (4)	C5M—C6M	1.513 (4)
O4K—C4K	1.437 (4)	C5M—H116	0.9800
O5H—C1H	1.412 (4)	C6I—H14A	0.9700
O5H—C5H	1.442 (4)	C6I—H14B	0.9700
O3D—C3D	1.432 (3)	C2B—H16	0.9800
O3D—H88	0.8200	C1O—H126	0.9800
O2D—C2D	1.420 (4)	C3N—C2N	1.511 (5)
O2D—H87	0.8200	C3N—H121	0.9800
O5P—C1P	1.411 (4)	C4J—C3J	1.511 (4)
O5P—C5P	1.447 (4)	C4J—C5J	1.536 (5)
O4O—C1N	1.407 (4)	C4J—H91	0.9800
O4O—C4O	1.436 (4)	C3J—C2J	1.525 (5)
O3M—C3M	1.422 (4)	C3J—H92	0.9800
O3M—H165	0.8200	C4A—C5A	1.511 (5)
O6G—C6G	1.426 (4)	C4A—H25	0.9800
O6G—H65	0.8200	C2N—H120	0.9800
O5I—C1I	1.403 (4)	C2J—C1J	1.524 (5)
O5I—C5I	1.430 (4)	C2J—H93	0.9800
O3P—C3P	1.422 (3)	C4F—C3F	1.512 (5)
O3P—H153	0.8200	C4F—C5F	1.533 (5)
O2B—C2B	1.429 (3)	C4F—H44	0.9800
O2B—H80	0.82 (6)	C2I—H143	0.9800
O4N—C4N	1.415 (4)	C5H—C6H	1.507 (5)
O4N—C1M	1.425 (4)	C5H—H33	0.9800
O4D—C1E	1.405 (4)	C5G—H40	0.9800
O4D—C4D	1.433 (4)	C6B—H77A	0.9700
O2L—C2L	1.413 (4)	C6B—H77B	0.9700
O2L—H169	0.8200	C1J—H94	0.9800
O5N—C1N	1.413 (4)	C5D—C6D	1.508 (5)
O5N—C5N	1.442 (4)	C5D—H5	0.9800
O5A—C1A	1.412 (4)	C6L—H16A	0.9700
O5A—C5A	1.433 (4)	C6L—H16B	0.9700
O2C—C2C	1.425 (3)	C1F—C2F	1.530 (6)
O2C—H84	0.8200	C1F—H47	0.9800
O1W—H18A	0.8592	C5K—C6K1	1.51 (2)
O1W—H18B	0.8584	C5K—C6K2	1.54 (3)
O5M—C1M	1.397 (4)	C5K—H102	0.9800
O5M—C5M	1.425 (4)	C6F—C5F	1.507 (5)
O2H—C2H	1.424 (4)	C6F—H60A	0.9700
O2H—H71	0.8200	C6F—H60B	0.9700
O5G—C1G	1.399 (4)	C6C—H81A	0.9700
O5G—C5G	1.438 (4)	C6C—H81B	0.9700
O6I—C6I	1.422 (4)	C3H—H31	0.9800
O6I—H148	0.8200	C6O—H15A	0.9700
O4E—C1F	1.404 (4)	C6O—H15B	0.9700
O4E—C4E	1.433 (4)	C1M—H112	0.9800
O2I—C2I	1.419 (4)	C2A—C1A	1.535 (4)
O2I—H149	0.8200	C2A—H23	0.9800
O3J—C3J	1.405 (4)	C1A—H22	0.9800
O3J—H176	0.8200	C6M—H16C	0.9700
O5F—C1F	1.406 (5)	C6M—H16D	0.9700
O5F—C5F	1.439 (4)	C1G—C2G	1.517 (4)
O2A—C2A	1.421 (4)	C1G—H36	0.9800
O2A—H73	0.8200	C2G—H37	0.9800
O2N—C2N	1.427 (4)	C5F—H43	0.9800
O2N—H162	0.8200	C5N—C6N	1.523 (5)
O6L—C6L	1.412 (5)	C5N—H123	0.9800
O6L—H168	0.8200	C5A—C6A1	1.519 (14)
O6C—C6C	1.410 (4)	C5A—C6A2	1.581 (15)
O6C—H82	0.8200	C5A—H26	0.9800
O3K—C3K	1.439 (4)	C5A—H26A	0.9800
O3I—C3I	1.433 (4)	C6P—H15C	0.9700
O3I—H150	0.8200	C6P—H15D	0.9700
O6F—C6F	1.422 (5)	C1E—C2E	1.539 (6)
O6F—H61	0.8200	C1E—H50	0.9800
O2G—C2G	1.408 (4)	C3F—C2F	1.525 (5)
O2G—H66	0.8200	C3F—H45	0.9800
O11W—H18C	0.8655	C4E—C3E	1.512 (6)
O11W—H18D	0.8649	C4E—C5E	1.521 (6)
O2W—H17A	0.8696	C4E—H53	0.9800
O2W—H17B	0.8698	C3E—C2E	1.515 (5)
O2J—C2J	1.410 (4)	C3E—H52	0.9800
O2J—H174	0.859 (12)	C2E—H51	0.9800
O6O—C6O	1.419 (5)	O6H1—C6H	1.484 (11)
O6O—H156	0.8200	O6H1—H69A	0.8200
O6M—C6M	1.419 (5)	O6H2—C6H	1.382 (10)
O6M—H164	0.8200	O6H2—H69B	0.8200
O3N—C3N	1.428 (4)	O6N—C6N	1.380 (6)
O3N—H161	0.8200	O6N—H160	0.8200
O2M—C2M	1.415 (4)	C6H—H68A	0.9700
O2M—H166	0.8200	C6H—H68B	0.9700
O6B—C6B	1.420 (4)	C6H—H68C	0.9700
O6B—H78	0.8200	C6H—H68D	0.9700
O3F—C3F	1.424 (5)	C5E—C6E2	1.478 (14)
O3F—H63	0.8200	C5E—C6E1	1.553 (15)
O5J—C1J	1.414 (4)	C2F—H46	0.9800
O5J—C5J	1.438 (7)	C6D—H85A	0.9700
O3E—C3E	1.434 (5)	C6D—H85B	0.9700
O3E—H59	0.8200	O6J2—C6J	1.370 (7)
O5E—C1E	1.422 (5)	O6J2—H96	0.8200
O5E—C5E	1.447 (5)	C5J—C6J	1.534 (7)
O2E—C2E	1.397 (6)	C5J—H90	0.9800
O2E—H57	0.8200	C6N—H15E	0.9700
O6D—C6D	1.402 (7)	C6N—H15F	0.9700
O6D—H86	0.8200	C6A2—O6A2	1.498 (17)
O2F—C2F	1.430 (5)	C6A2—H74A	0.9700
O2F—H62	0.8200	C6A2—H74B	0.9700
O6P—C6P	1.424 (7)	O6A2—H76B	1.19 (18)
O6P—H152	0.8200	O6A1—C6A1	1.382 (14)
C3O—C4O	1.510 (4)	O6A1—H76A	0.8200
C3O—C2O	1.514 (4)	C6A1—H74C	0.9700
C3O—H128	0.9800	C6A1—H74D	0.9700
C2L—C3L	1.519 (4)	C6J—O6J1	1.465 (8)
C2L—C1L	1.524 (4)	C6J—H95A	0.9700
C2L—H108	0.9800	C6J—H95B	0.9700
C1K—C2K	1.525 (4)	C6J—H95C	0.9700
C1K—H98	0.9800	C6J—H95D	0.9700
C4B—C5B	1.525 (4)	O6E1—C6E1	1.343 (17)
C4B—C3B	1.532 (4)	O6E1—H3A	0.8200
C4B—H18	0.9800	C6E1—H58A	0.9700
C1L—H109	0.9800	C6E1—H58B	0.9700
C1B—C2B	1.545 (4)	O6E2—C6E2	1.462 (15)
C1B—H15	0.9800	O6E2—H3B	0.8200
C5I—C6I	1.503 (4)	C6E2—H58C	0.9700
C5I—C4I	1.532 (4)	C6E2—H58D	0.9700
C5I—H140	0.9800	O3W—H2WA	0.8510
C1H—C2H	1.522 (4)	O3W—H2WB	0.8500
C1H—H29	0.9800	O5W—H3WA	0.8509
C2K—C3K	1.520 (4)	O5W—H3WB	0.8493
C2K—H99	0.9800	O4W—H4WA	0.8485
C3M—C4M	1.508 (4)	O4W—H4WB	0.8501
C3M—C2M	1.531 (5)	O6W—H5WA	0.8485
C3M—H114	0.9800	O6W—H5WB	0.8513
C4K—C3K	1.523 (4)	O9W—H6WA	0.8516
C4K—C5K	1.530 (4)	O9W—H6WB	0.8492
C4K—H101	0.9800	O7W—H7WA	0.8500
C4L—C3L	1.514 (4)	O7W—H7WB	0.8500
C4L—C5L	1.528 (4)	O8W—H8WA	0.8304
C4L—H106	0.9800	O8W—H8WB	0.8507
C4C—C5C	1.527 (4)	O6K1—C6K1	1.416 (8)
C4C—C3C	1.535 (4)	O6K1—H1D	0.8200
C4C—H11	0.9800	C6K1—H6A	0.9700
C1I—C2I	1.529 (4)	C6K1—H6B	0.9700
C1I—H144	0.9800	O10W—H0AA	0.8505
C5L—C6L	1.523 (4)	O10W—H0AB	0.8498
C5L—H105	0.9800	O6J1—H96A	0.8200
C3L—H107	0.9800	O6K2—C6K2	1.414 (9)
C1D—C2D	1.531 (4)	O6K2—H1E	0.8200
C1D—H1	0.9800	C6K2—H6AA	0.9700
C5B—C6B	1.520 (4)	C6K2—H6AB	0.9700
			
O2W—Li1—O1W	107.3 (3)	C2D—C3D—H3	109.7
O2W—Li1—O2I	106.3 (3)	O3I—C3I—C4I	108.3 (3)
O1W—Li1—O2I	121.0 (3)	O3I—C3I—C2I	110.6 (3)
O2W—Li1—O3A	118.8 (3)	C4I—C3I—C2I	108.6 (3)
O1W—Li1—O3A	100.1 (3)	O3I—C3I—H142	109.7
O2I—Li1—O3A	104.3 (3)	C4I—C3I—H142	109.7
O2W—Li1—H17B	20.4	C2I—C3I—H142	109.7
O1W—Li1—H17B	121.9	O2M—C2M—C1M	111.2 (3)
O2I—Li1—H17B	105.4	O2M—C2M—C3M	112.3 (3)
O3A—Li1—H17B	100.2	C1M—C2M—C3M	111.6 (3)
O2J—Li2—O11W	107.0 (4)	O2M—C2M—H113	107.2
O2J—Li2—O3E^i^	119.8 (4)	C1M—C2M—H113	107.2
O11W—Li2—O3E^i^	114.8 (4)	C3M—C2M—H113	107.2
O2J—Li2—O3K	98.3 (4)	O4O—C1N—O5N	112.0 (3)
O11W—Li2—O3K	98.1 (3)	O4O—C1N—C2N	107.9 (3)
O3E^i^—Li2—O3K	115.7 (4)	O5N—C1N—C2N	109.8 (2)
C1H—O4G—C4G	117.4 (2)	O4O—C1N—H119	109.0
C1C—O4B—C4B	118.8 (2)	O5N—C1N—H119	109.0
C1P—O4I—C4I	116.5 (2)	C2N—C1N—H119	109.0
C1L—O5L—C5L	115.5 (2)	O5M—C5M—C6M	102.5 (3)
C1L—O4M—C4M	116.5 (2)	O5M—C5M—C4M	110.9 (3)
C1D—O5D—C5D	116.5 (2)	C6M—C5M—C4M	111.3 (3)
C1D—O4C—C4C	115.8 (2)	O5M—C5M—H116	110.6
C1K—O4L—C4L	118.0 (2)	C6M—C5M—H116	110.6
C2K—O2K—H171	109.5	C4M—C5M—H116	110.6
C1B—O5B—C5B	113.8 (2)	O6I—C6I—C5I	111.2 (3)
C1O—O4P—C4P	115.8 (2)	O6I—C6I—H14A	109.4
C2P—O2P—H154	109.5	C5I—C6I—H14A	109.4
C3B—O3B—H79	109.5	O6I—C6I—H14B	109.4
C3C—O3C—H83	109.5	C5I—C6I—H14B	109.4
C1C—O5C—C5C	113.6 (2)	H14A—C6I—H14B	108.0
C1A—O4H—C4H	116.0 (2)	O2B—C2B—C3B	111.8 (2)
C4F—O4F—C1G	117.5 (3)	O2B—C2B—C1B	109.6 (2)
C1B—O4A—C4A	116.1 (2)	C3B—C2B—C1B	112.2 (2)
C3L—O3L—H170	109.5	O2B—C2B—H16	107.7
C1O—O5O—C5O	115.0 (2)	C3B—C2B—H16	107.7
C1K—O5K—C5K	113.6 (2)	C1B—C2B—H16	107.7
C1I—O4J—C4J	116.3 (2)	O5O—C1O—O4P	111.7 (2)
C3A—O3A—Li1	125.3 (2)	O5O—C1O—C2O	111.4 (2)
C3H—O3H—H70	109.5	O4P—C1O—C2O	109.6 (2)
C3O—O3O—H157	109.5	O5O—C1O—H126	108.0
C2O—O2O—H158	109.5	O4P—C1O—H126	108.0
C3G—O3G—H67	109.5	C2O—C1O—H126	108.0
C1J—O4K—C4K	116.9 (3)	O3N—C3N—C2N	114.5 (3)
C1H—O5H—C5H	114.3 (2)	O3N—C3N—C4N	108.9 (3)
C3D—O3D—H88	109.5	C2N—C3N—C4N	109.7 (3)
C2D—O2D—H87	109.5	O3N—C3N—H121	107.9
C1P—O5P—C5P	113.5 (2)	C2N—C3N—H121	107.9
C1N—O4O—C4O	119.1 (2)	C4N—C3N—H121	107.9
C3M—O3M—H165	109.5	O4J—C4J—C3J	108.1 (3)
C6G—O6G—H65	109.5	O4J—C4J—C5J	110.1 (4)
C1I—O5I—C5I	115.4 (2)	C3J—C4J—C5J	109.7 (3)
C3P—O3P—H153	109.5	O4J—C4J—H91	109.7
C2B—O2B—H80	109.5	C3J—C4J—H91	109.7
C4N—O4N—C1M	116.3 (2)	C5J—C4J—H91	109.7
C1E—O4D—C4D	117.9 (2)	O3J—C3J—C4J	110.2 (3)
C2L—O2L—H169	109.5	O3J—C3J—C2J	112.7 (3)
C1N—O5N—C5N	113.2 (2)	C4J—C3J—C2J	107.0 (3)
C1A—O5A—C5A	114.6 (2)	O3J—C3J—H92	108.9
C2C—O2C—H84	109.5	C4J—C3J—H92	108.9
Li1—O1W—H18A	110.6	C2J—C3J—H92	108.9
Li1—O1W—H18B	109.8	O4A—C4A—C5A	110.8 (3)
H18A—O1W—H18B	103.8	O4A—C4A—C3A	106.8 (2)
C1M—O5M—C5M	116.6 (2)	C5A—C4A—C3A	110.0 (2)
C2H—O2H—H71	109.5	O4A—C4A—H25	109.7
C1G—O5G—C5G	113.0 (2)	C5A—C4A—H25	109.7
C6I—O6I—H148	109.5	C3A—C4A—H25	109.7
C1F—O4E—C4E	118.5 (3)	O2N—C2N—C3N	111.6 (3)
C2I—O2I—Li1	121.0 (3)	O2N—C2N—C1N	109.3 (3)
C2I—O2I—H149	109.5	C3N—C2N—C1N	109.8 (3)
Li1—O2I—H149	129.5	O2N—C2N—H120	108.7
C3J—O3J—H176	109.5	C3N—C2N—H120	108.7
C1F—O5F—C5F	113.6 (3)	C1N—C2N—H120	108.7
C2A—O2A—H73	109.5	O2J—C2J—C1J	109.3 (3)
C2N—O2N—H162	109.5	O2J—C2J—C3J	112.6 (3)
C6L—O6L—H168	109.5	C1J—C2J—C3J	112.2 (3)
C6C—O6C—H82	109.5	O2J—C2J—H93	107.5
C3K—O3K—Li2	127.0 (3)	C1J—C2J—H93	107.5
C3I—O3I—H150	109.5	C3J—C2J—H93	107.5
C6F—O6F—H61	109.5	O4F—C4F—C3F	106.3 (3)
C2G—O2G—H66	109.5	O4F—C4F—C5F	110.7 (3)
Li2—O11W—H18C	110.8	C3F—C4F—C5F	110.1 (3)
Li2—O11W—H18D	109.9	O4F—C4F—H44	109.9
H18C—O11W—H18D	103.5	C3F—C4F—H44	109.9
Li1—O2W—H17A	111.1	C5F—C4F—H44	109.9
Li1—O2W—H17B	110.4	O2I—C2I—C3I	112.0 (3)
H17A—O2W—H17B	103.2	O2I—C2I—C1I	111.0 (3)
C2J—O2J—Li2	132.0 (3)	C3I—C2I—C1I	109.9 (3)
C2J—O2J—H174	108.9 (17)	O2I—C2I—H143	107.9
Li2—O2J—H174	116.0 (17)	C3I—C2I—H143	107.9
C6O—O6O—H156	109.5	C1I—C2I—H143	107.9
C6M—O6M—H164	109.5	O5H—C5H—C6H	107.5 (3)
C3N—O3N—H161	109.5	O5H—C5H—C4H	109.0 (3)
C2M—O2M—H166	109.5	C6H—C5H—C4H	113.1 (3)
C6B—O6B—H78	109.5	O5H—C5H—H33	109.1
C3F—O3F—H63	109.5	C6H—C5H—H33	109.1
C1J—O5J—C5J	115.0 (3)	C4H—C5H—H33	109.1
C3E—O3E—Li2^ii^	132.2 (4)	O5G—C5G—C6G	106.3 (2)
C3E—O3E—H59	109.5	O5G—C5G—C4G	109.6 (2)
Li2^ii^—O3E—H59	115.1	C6G—C5G—C4G	113.4 (3)
C1E—O5E—C5E	113.6 (3)	O5G—C5G—H40	109.1
C2E—O2E—H57	109.5	C6G—C5G—H40	109.1
C6D—O6D—H86	109.5	C4G—C5G—H40	109.1
C2F—O2F—H62	109.5	O6B—C6B—C5B	110.9 (3)
C6P—O6P—H152	109.5	O6B—C6B—H77A	109.5
O3O—C3O—C4O	110.9 (2)	C5B—C6B—H77A	109.5
O3O—C3O—C2O	107.5 (2)	O6B—C6B—H77B	109.5
C4O—C3O—C2O	110.5 (2)	C5B—C6B—H77B	109.5
O3O—C3O—H128	109.3	H77A—C6B—H77B	108.0
C4O—C3O—H128	109.3	O4K—C1J—O5J	110.6 (3)
C2O—C3O—H128	109.3	O4K—C1J—C2J	109.4 (3)
O2L—C2L—C3L	112.2 (2)	O5J—C1J—C2J	109.1 (3)
O2L—C2L—C1L	111.1 (2)	O4K—C1J—H94	109.3
C3L—C2L—C1L	111.3 (2)	O5J—C1J—H94	109.3
O2L—C2L—H108	107.3	C2J—C1J—H94	109.3
C3L—C2L—H108	107.3	O5D—C5D—C6D	105.7 (3)
C1L—C2L—H108	107.3	O5D—C5D—C4D	108.6 (2)
O4L—C1K—O5K	111.4 (2)	C6D—C5D—C4D	112.9 (3)
O4L—C1K—C2K	108.8 (2)	O5D—C5D—H5	109.8
O5K—C1K—C2K	109.4 (2)	C6D—C5D—H5	109.8
O4L—C1K—H98	109.1	C4D—C5D—H5	109.8
O5K—C1K—H98	109.1	O6L—C6L—C5L	112.4 (3)
C2K—C1K—H98	109.1	O6L—C6L—H16A	109.1
O4B—C4B—C5B	108.3 (2)	C5L—C6L—H16A	109.1
O4B—C4B—C3B	105.4 (2)	O6L—C6L—H16B	109.1
C5B—C4B—C3B	112.2 (2)	C5L—C6L—H16B	109.1
O4B—C4B—H18	110.3	H16A—C6L—H16B	107.8
C5B—C4B—H18	110.3	O4E—C1F—O5F	110.1 (3)
C3B—C4B—H18	110.3	O4E—C1F—C2F	108.7 (3)
O5L—C1L—O4M	111.6 (2)	O5F—C1F—C2F	111.4 (3)
O5L—C1L—C2L	111.1 (2)	O4E—C1F—H47	108.9
O4M—C1L—C2L	108.6 (2)	O5F—C1F—H47	108.9
O5L—C1L—H109	108.5	C2F—C1F—H47	108.9
O4M—C1L—H109	108.5	O5K—C5K—C6K1	109.8 (10)
C2L—C1L—H109	108.5	O5K—C5K—C4K	109.0 (3)
O4A—C1B—O5B	110.4 (2)	C6K1—C5K—C4K	115.0 (7)
O4A—C1B—C2B	109.6 (2)	O5K—C5K—C6K2	99.9 (11)
O5B—C1B—C2B	109.7 (2)	C4K—C5K—C6K2	108.8 (7)
O4A—C1B—H15	109.0	O5K—C5K—H102	112.8
O5B—C1B—H15	109.0	C4K—C5K—H102	112.8
C2B—C1B—H15	109.0	C6K2—C5K—H102	112.8
O5I—C5I—C6I	106.2 (2)	O6F—C6F—C5F	112.9 (3)
O5I—C5I—C4I	110.1 (2)	O6F—C6F—H60A	109.0
C6I—C5I—C4I	113.2 (3)	C5F—C6F—H60A	109.0
O5I—C5I—H140	109.1	O6F—C6F—H60B	109.0
C6I—C5I—H140	109.1	C5F—C6F—H60B	109.0
C4I—C5I—H140	109.1	H60A—C6F—H60B	107.8
O5H—C1H—O4G	111.7 (2)	O6C—C6C—C5C	110.9 (3)
O5H—C1H—C2H	110.4 (3)	O6C—C6C—H81A	109.5
O4G—C1H—C2H	108.2 (2)	C5C—C6C—H81A	109.5
O5H—C1H—H29	108.8	O6C—C6C—H81B	109.5
O4G—C1H—H29	108.8	C5C—C6C—H81B	109.5
C2H—C1H—H29	108.8	H81A—C6C—H81B	108.0
O2K—C2K—C3K	112.2 (2)	O3H—C3H—C2H	109.7 (2)
O2K—C2K—C1K	109.2 (2)	O3H—C3H—C4H	111.4 (2)
C3K—C2K—C1K	111.7 (2)	C2H—C3H—C4H	107.4 (2)
O2K—C2K—H99	107.9	O3H—C3H—H31	109.4
C3K—C2K—H99	107.9	C2H—C3H—H31	109.4
C1K—C2K—H99	107.9	C4H—C3H—H31	109.4
O3M—C3M—C4M	107.6 (2)	O6O—C6O—C5O	112.1 (3)
O3M—C3M—C2M	110.8 (3)	O6O—C6O—H15A	109.2
C4M—C3M—C2M	108.0 (2)	C5O—C6O—H15A	109.2
O3M—C3M—H114	110.1	O6O—C6O—H15B	109.2
C4M—C3M—H114	110.1	C5O—C6O—H15B	109.2
C2M—C3M—H114	110.1	H15A—C6O—H15B	107.9
O4K—C4K—C3K	106.8 (2)	O5M—C1M—O4N	110.6 (3)
O4K—C4K—C5K	110.3 (3)	O5M—C1M—C2M	111.6 (3)
C3K—C4K—C5K	111.1 (2)	O4N—C1M—C2M	108.7 (3)
O4K—C4K—H101	109.5	O5M—C1M—H112	108.6
C3K—C4K—H101	109.5	O4N—C1M—H112	108.6
C5K—C4K—H101	109.5	C2M—C1M—H112	108.6
O4L—C4L—C3L	107.7 (2)	O2A—C2A—C3A	109.2 (2)
O4L—C4L—C5L	108.6 (2)	O2A—C2A—C1A	111.5 (2)
C3L—C4L—C5L	110.8 (3)	C3A—C2A—C1A	111.6 (3)
O4L—C4L—H106	109.9	O2A—C2A—H23	108.2
C3L—C4L—H106	109.9	C3A—C2A—H23	108.2
C5L—C4L—H106	109.9	C1A—C2A—H23	108.2
O4C—C4C—C5C	110.5 (2)	O5A—C1A—O4H	110.3 (3)
O4C—C4C—C3C	108.2 (2)	O5A—C1A—C2A	111.2 (2)
C5C—C4C—C3C	110.8 (2)	O4H—C1A—C2A	110.2 (2)
O4C—C4C—H11	109.1	O5A—C1A—H22	108.4
C5C—C4C—H11	109.1	O4H—C1A—H22	108.4
C3C—C4C—H11	109.1	C2A—C1A—H22	108.4
O5I—C1I—O4J	110.7 (2)	O6M—C6M—C5M	110.2 (3)
O5I—C1I—C2I	110.9 (3)	O6M—C6M—H16C	109.6
O4J—C1I—C2I	109.0 (3)	C5M—C6M—H16C	109.6
O5I—C1I—H144	108.7	O6M—C6M—H16D	109.6
O4J—C1I—H144	108.7	C5M—C6M—H16D	109.6
C2I—C1I—H144	108.7	H16C—C6M—H16D	108.1
O5L—C5L—C6L	105.2 (2)	O5G—C1G—O4F	109.4 (2)
O5L—C5L—C4L	109.9 (2)	O5G—C1G—C2G	111.5 (3)
C6L—C5L—C4L	113.8 (3)	O4F—C1G—C2G	108.5 (3)
O5L—C5L—H105	109.3	O5G—C1G—H36	109.1
C6L—C5L—H105	109.3	O4F—C1G—H36	109.1
C4L—C5L—H105	109.3	C2G—C1G—H36	109.1
O3L—C3L—C4L	110.4 (2)	O2G—C2G—C1G	109.6 (3)
O3L—C3L—C2L	108.5 (2)	O2G—C2G—C3G	111.7 (3)
C4L—C3L—C2L	110.2 (2)	C1G—C2G—C3G	109.4 (3)
O3L—C3L—H107	109.3	O2G—C2G—H37	108.7
C4L—C3L—H107	109.3	C1G—C2G—H37	108.7
C2L—C3L—H107	109.3	C3G—C2G—H37	108.7
O5D—C1D—O4C	111.5 (2)	O5F—C5F—C6F	109.6 (3)
O5D—C1D—C2D	111.0 (2)	O5F—C5F—C4F	106.9 (3)
O4C—C1D—C2D	108.3 (2)	C6F—C5F—C4F	115.4 (3)
O5D—C1D—H1	108.7	O5F—C5F—H43	108.2
O4C—C1D—H1	108.7	C6F—C5F—H43	108.2
C2D—C1D—H1	108.7	C4F—C5F—H43	108.2
O5B—C5B—C6B	106.7 (2)	O5N—C5N—C6N	105.5 (3)
O5B—C5B—C4B	109.4 (2)	O5N—C5N—C4N	109.3 (3)
C6B—C5B—C4B	112.2 (3)	C6N—C5N—C4N	111.9 (3)
O5B—C5B—H19	109.5	O5N—C5N—H123	110.0
C6B—C5B—H19	109.5	C6N—C5N—H123	110.0
C4B—C5B—H19	109.5	C4N—C5N—H123	110.0
O4O—C4O—C3O	106.0 (2)	O5A—C5A—C4A	107.8 (3)
O4O—C4O—C5O	110.4 (2)	O5A—C5A—C6A1	112.0 (6)
C3O—C4O—C5O	111.4 (2)	C4A—C5A—C6A1	117.7 (6)
O4O—C4O—H129	109.7	O5A—C5A—C6A2	100.6 (6)
C3O—C4O—H129	109.7	C4A—C5A—C6A2	107.4 (6)
C5O—C4O—H129	109.7	O5A—C5A—H26	113.4
O3B—C3B—C2B	111.0 (2)	C4A—C5A—H26	113.4
O3B—C3B—C4B	110.4 (2)	C6A2—C5A—H26	113.4
C2B—C3B—C4B	110.8 (2)	O5A—C5A—H26A	106.2
O3B—C3B—H17	108.2	C4A—C5A—H26A	106.2
C2B—C3B—H17	108.2	C6A1—C5A—H26A	106.2
C4B—C3B—H17	108.2	O6P—C6P—C5P	110.2 (4)
O3K—C3K—C2K	108.7 (2)	O6P—C6P—H15C	109.6
O3K—C3K—C4K	110.7 (2)	C5P—C6P—H15C	109.6
C2K—C3K—C4K	110.5 (2)	O6P—C6P—H15D	109.6
O3K—C3K—H100	109.0	C5P—C6P—H15D	109.6
C2K—C3K—H100	109.0	H15C—C6P—H15D	108.1
C4K—C3K—H100	109.0	O4D—C1E—O5E	110.3 (3)
O4I—C4I—C3I	106.8 (2)	O4D—C1E—C2E	109.6 (3)
O4I—C4I—C5I	109.9 (2)	O5E—C1E—C2E	111.4 (3)
C3I—C4I—C5I	110.4 (3)	O4D—C1E—H50	108.5
O4I—C4I—H141	109.9	O5E—C1E—H50	108.5
C3I—C4I—H141	109.9	C2E—C1E—H50	108.5
C5I—C4I—H141	109.9	O3F—C3F—C4F	110.2 (3)
O4H—C4H—C5H	111.0 (3)	O3F—C3F—C2F	109.8 (3)
O4H—C4H—C3H	108.2 (2)	C4F—C3F—C2F	109.9 (3)
C5H—C4H—C3H	109.9 (3)	O3F—C3F—H45	109.0
O4H—C4H—H32	109.2	C4F—C3F—H45	109.0
C5H—C4H—H32	109.2	C2F—C3F—H45	109.0
C3H—C4H—H32	109.2	O4E—C4E—C3E	107.1 (3)
O5O—C5O—C6O	105.0 (3)	O4E—C4E—C5E	107.9 (3)
O5O—C5O—C4O	109.9 (2)	C3E—C4E—C5E	111.5 (3)
C6O—C5O—C4O	114.3 (3)	O4E—C4E—H53	110.1
O5O—C5O—H130	109.2	C3E—C4E—H53	110.1
C6O—C5O—H130	109.2	C5E—C4E—H53	110.1
C4O—C5O—H130	109.2	O3E—C3E—C4E	111.0 (3)
O4P—C4P—C5P	109.9 (2)	O3E—C3E—C2E	108.4 (3)
O4P—C4P—C3P	108.2 (2)	C4E—C3E—C2E	111.6 (4)
C5P—C4P—C3P	110.3 (2)	O3E—C3E—H52	108.6
O4P—C4P—H136	109.5	C4E—C3E—H52	108.6
C5P—C4P—H136	109.5	C2E—C3E—H52	108.6
C3P—C4P—H136	109.5	O2E—C2E—C3E	112.9 (4)
O5C—C5C—C6C	106.2 (3)	O2E—C2E—C1E	107.5 (3)
O5C—C5C—C4C	108.5 (2)	C3E—C2E—C1E	110.0 (3)
C6C—C5C—C4C	114.1 (3)	O2E—C2E—H51	108.8
O5C—C5C—H12	109.3	C3E—C2E—H51	108.8
C6C—C5C—H12	109.3	C1E—C2E—H51	108.8
C4C—C5C—H12	109.3	C6H—O6H1—H69A	109.5
O2D—C2D—C3D	111.1 (2)	C6H—O6H2—H69B	109.5
O2D—C2D—C1D	112.1 (2)	C6N—O6N—H160	109.5
C3D—C2D—C1D	111.5 (2)	O6H2—C6H—C5H	112.3 (5)
O2D—C2D—H2	107.3	O6H1—C6H—C5H	108.9 (5)
C3D—C2D—H2	107.3	O6H1—C6H—H68A	109.9
C1D—C2D—H2	107.3	C5H—C6H—H68A	109.9
O5P—C1P—O4I	110.6 (3)	O6H1—C6H—H68B	109.9
O5P—C1P—C2P	111.1 (3)	C5H—C6H—H68B	109.9
O4I—C1P—C2P	108.3 (2)	H68A—C6H—H68B	108.3
O5P—C1P—H133	108.9	O6H2—C6H—H68C	109.1
O4I—C1P—H133	108.9	C5H—C6H—H68C	109.1
C2P—C1P—H133	108.9	O6H2—C6H—H68D	109.1
O2P—C2P—C3P	110.5 (2)	C5H—C6H—H68D	109.1
O2P—C2P—C1P	110.9 (2)	H68C—C6H—H68D	107.9
C3P—C2P—C1P	111.7 (2)	O5E—C5E—C6E2	101.9 (7)
O2P—C2P—H134	107.9	O5E—C5E—C4E	107.8 (4)
C3P—C2P—H134	107.9	C6E2—C5E—C4E	106.7 (7)
C1P—C2P—H134	107.9	O5E—C5E—C6E1	112.6 (6)
O4N—C4N—C5N	111.4 (3)	C4E—C5E—C6E1	119.9 (7)
O4N—C4N—C3N	107.2 (3)	O2F—C2F—C3F	112.2 (4)
C5N—C4N—C3N	111.4 (2)	O2F—C2F—C1F	109.1 (3)
O4N—C4N—H122	108.9	C3F—C2F—C1F	111.3 (3)
C5N—C4N—H122	108.9	O2F—C2F—H46	108.0
C3N—C4N—H122	108.9	C3F—C2F—H46	108.0
O3C—C3C—C2C	111.5 (2)	C1F—C2F—H46	108.0
O3C—C3C—C4C	107.5 (2)	O6D—C6D—C5D	111.8 (3)
C2C—C3C—C4C	108.4 (2)	O6D—C6D—H85A	109.3
O3C—C3C—H10	109.8	C5D—C6D—H85A	109.3
C2C—C3C—H10	109.8	O6D—C6D—H85B	109.3
C4C—C3C—H10	109.8	C5D—C6D—H85B	109.3
O2C—C2C—C3C	112.9 (2)	H85A—C6D—H85B	107.9
O2C—C2C—C1C	109.5 (2)	C6J—O6J2—H96	109.5
C3C—C2C—C1C	110.4 (2)	O5J—C5J—C6J	116.8 (4)
O2C—C2C—H9	107.9	O5J—C5J—C4J	108.5 (5)
C3C—C2C—H9	107.9	C6J—C5J—C4J	119.0 (4)
C1C—C2C—H9	107.9	O5J—C5J—H90	103.4
O3P—C3P—C2P	108.8 (2)	C6J—C5J—H90	103.4
O3P—C3P—C4P	111.0 (2)	C4J—C5J—H90	103.4
C2P—C3P—C4P	110.0 (2)	O6N—C6N—C5N	114.0 (4)
O3P—C3P—H135	109.0	O6N—C6N—H15E	108.7
C2P—C3P—H135	109.0	C5N—C6N—H15E	108.7
C4P—C3P—H135	109.0	O6N—C6N—H15F	108.7
O4D—C4D—C3D	108.0 (2)	C5N—C6N—H15F	108.7
O4D—C4D—C5D	110.1 (2)	H15E—C6N—H15F	107.6
C3D—C4D—C5D	109.4 (3)	O6A2—C6A2—C5A	116.4 (10)
O4D—C4D—H4	109.8	O6A2—C6A2—H74A	108.2
C3D—C4D—H4	109.8	C5A—C6A2—H74A	108.2
C5D—C4D—H4	109.8	O6A2—C6A2—H74B	108.2
O3A—C3A—C2A	107.4 (2)	C5A—C6A2—H74B	108.2
O3A—C3A—C4A	110.4 (2)	H74A—C6A2—H74B	107.3
C2A—C3A—C4A	112.2 (2)	C6A2—O6A2—H76B	109.5
O3A—C3A—H24	108.9	C6A1—O6A1—H76A	109.5
C2A—C3A—H24	108.9	O6A1—C6A1—C5A	115.2 (9)
C4A—C3A—H24	108.9	O6A1—C6A1—H74C	108.5
O2H—C2H—C3H	112.2 (2)	C5A—C6A1—H74C	108.5
O2H—C2H—C1H	111.8 (3)	O6A1—C6A1—H74D	108.5
C3H—C2H—C1H	109.2 (2)	C5A—C6A1—H74D	108.5
O2H—C2H—H30	107.8	H74C—C6A1—H74D	107.5
C3H—C2H—H30	107.8	O6J2—C6J—C5J	91.1 (6)
C1H—C2H—H30	107.8	O6J1—C6J—C5J	110.4 (7)
O5P—C5P—C6P	107.1 (3)	O6J2—C6J—H95A	113.4
O5P—C5P—C4P	107.8 (3)	C5J—C6J—H95A	113.4
C6P—C5P—C4P	112.7 (3)	O6J2—C6J—H95B	113.4
O5P—C5P—H137	109.7	C5J—C6J—H95B	113.4
C6P—C5P—H137	109.7	H95A—C6J—H95B	110.7
C4P—C5P—H137	109.7	O6J1—C6J—H95C	109.6
O3G—C3G—C4G	112.4 (2)	C5J—C6J—H95C	109.6
O3G—C3G—C2G	112.2 (2)	O6J1—C6J—H95D	109.6
C4G—C3G—C2G	109.2 (2)	C5J—C6J—H95D	109.6
O3G—C3G—H38	107.6	H95C—C6J—H95D	108.1
C4G—C3G—H38	107.6	C6E1—O6E1—H3A	109.5
C2G—C3G—H38	107.6	O6E1—C6E1—C5E	111.2 (13)
O4M—C4M—C3M	109.1 (2)	O6E1—C6E1—H58A	109.4
O4M—C4M—C5M	111.1 (2)	C5E—C6E1—H58A	109.4
C3M—C4M—C5M	109.6 (2)	O6E1—C6E1—H58B	109.4
O4M—C4M—H115	109.0	C5E—C6E1—H58B	109.4
C3M—C4M—H115	109.0	H58A—C6E1—H58B	108.0
C5M—C4M—H115	109.0	C6E2—O6E2—H3B	109.5
O4G—C4G—C3G	106.7 (2)	O6E2—C6E2—C5E	107.0 (10)
O4G—C4G—C5G	109.1 (2)	O6E2—C6E2—H58C	110.3
C3G—C4G—C5G	110.1 (2)	C5E—C6E2—H58C	110.3
O4G—C4G—H39	110.3	O6E2—C6E2—H58D	110.3
C3G—C4G—H39	110.3	C5E—C6E2—H58D	110.3
C5G—C4G—H39	110.3	H58C—C6E2—H58D	108.6
O6G—C6G—C5G	110.3 (3)	H2WA—O3W—H2WB	104.6
O6G—C6G—H64A	109.6	H3WA—O5W—H3WB	104.4
C5G—C6G—H64A	109.6	H4WA—O4W—H4WB	104.7
O6G—C6G—H64B	109.6	H5WA—O6W—H5WB	109.5
C5G—C6G—H64B	109.6	H6WA—O9W—H6WB	104.4
H64A—C6G—H64B	108.1	H7WA—O7W—H7WB	104.5
O2O—C2O—C3O	112.8 (3)	H8WA—O8W—H8WB	132.5
O2O—C2O—C1O	109.6 (2)	C6K1—O6K1—H1D	109.5
C3O—C2O—C1O	110.4 (2)	O6K1—C6K1—C5K	110.9 (16)
O2O—C2O—H127	108.0	O6K1—C6K1—H6A	109.5
C3O—C2O—H127	108.0	C5K—C6K1—H6A	109.5
C1O—C2O—H127	108.0	O6K1—C6K1—H6B	109.5
O5C—C1C—O4B	109.4 (2)	C5K—C6K1—H6B	109.5
O5C—C1C—C2C	110.3 (2)	H6A—C6K1—H6B	108.1
O4B—C1C—C2C	108.5 (2)	H0AA—O10W—H0AB	104.4
O5C—C1C—H8	109.5	C6J—O6J1—H96A	109.5
O4B—C1C—H8	109.5	C6K2—O6K2—H1E	109.5
C2C—C1C—H8	109.5	O6K2—C6K2—C5K	113.2 (19)
O3D—C3D—C4D	110.3 (2)	O6K2—C6K2—H6AA	108.9
O3D—C3D—C2D	108.7 (2)	C5K—C6K2—H6AA	108.9
C4D—C3D—C2D	108.9 (2)	O6K2—C6K2—H6AB	108.9
O3D—C3D—H3	109.7	C5K—C6K2—H6AB	108.9
C4D—C3D—H3	109.7	H6AA—C6K2—H6AB	107.7
			
C4L—O4L—C1K—O5K	106.5 (3)	C5J—C4J—C3J—O3J	−178.8 (4)
C4L—O4L—C1K—C2K	−132.8 (2)	O4J—C4J—C3J—C2J	178.3 (3)
C5K—O5K—C1K—O4L	58.3 (3)	C5J—C4J—C3J—C2J	58.3 (5)
C5K—O5K—C1K—C2K	−62.0 (3)	C1B—O4A—C4A—C5A	−136.4 (3)
C1C—O4B—C4B—C5B	−108.3 (3)	C1B—O4A—C4A—C3A	103.8 (3)
C1C—O4B—C4B—C3B	131.4 (2)	O3A—C3A—C4A—O4A	−67.0 (3)
C5L—O5L—C1L—O4M	64.4 (3)	C2A—C3A—C4A—O4A	173.2 (3)
C5L—O5L—C1L—C2L	−57.0 (3)	O3A—C3A—C4A—C5A	172.7 (3)
C4M—O4M—C1L—O5L	106.0 (3)	C2A—C3A—C4A—C5A	52.9 (4)
C4M—O4M—C1L—C2L	−131.2 (2)	O3N—C3N—C2N—O2N	62.0 (4)
O2L—C2L—C1L—O5L	178.5 (2)	C4N—C3N—C2N—O2N	−175.3 (3)
C3L—C2L—C1L—O5L	52.6 (3)	O3N—C3N—C2N—C1N	−176.7 (3)
O2L—C2L—C1L—O4M	55.4 (3)	C4N—C3N—C2N—C1N	−53.9 (3)
C3L—C2L—C1L—O4M	−70.5 (3)	O4O—C1N—C2N—O2N	59.1 (3)
C4A—O4A—C1B—O5B	107.7 (3)	O5N—C1N—C2N—O2N	−178.6 (3)
C4A—O4A—C1B—C2B	−131.4 (2)	O4O—C1N—C2N—C3N	−63.7 (3)
C5B—O5B—C1B—O4A	59.8 (3)	O5N—C1N—C2N—C3N	58.6 (3)
C5B—O5B—C1B—C2B	−61.0 (3)	Li2—O2J—C2J—C1J	−78.2 (5)
C1I—O5I—C5I—C6I	179.8 (3)	Li2—O2J—C2J—C3J	47.2 (5)
C1I—O5I—C5I—C4I	56.9 (3)	O3J—C3J—C2J—O2J	58.3 (3)
C5H—O5H—C1H—O4G	61.7 (3)	C4J—C3J—C2J—O2J	179.6 (3)
C5H—O5H—C1H—C2H	−58.7 (3)	O3J—C3J—C2J—C1J	−177.9 (3)
C4G—O4G—C1H—O5H	108.7 (3)	C4J—C3J—C2J—C1J	−56.6 (3)
C4G—O4G—C1H—C2H	−129.6 (3)	C1G—O4F—C4F—C3F	139.4 (3)
O4L—C1K—C2K—O2K	58.0 (3)	C1G—O4F—C4F—C5F	−100.9 (3)
O5K—C1K—C2K—O2K	179.9 (2)	Li1—O2I—C2I—C3I	−101.9 (3)
O4L—C1K—C2K—C3K	−66.6 (3)	Li1—O2I—C2I—C1I	134.9 (3)
O5K—C1K—C2K—C3K	55.3 (3)	O3I—C3I—C2I—O2I	61.3 (3)
C1J—O4K—C4K—C3K	131.4 (3)	C4I—C3I—C2I—O2I	−179.9 (2)
C1J—O4K—C4K—C5K	−107.8 (3)	O3I—C3I—C2I—C1I	−174.8 (3)
C1K—O4L—C4L—C3L	109.2 (3)	C4I—C3I—C2I—C1I	−56.0 (3)
C1K—O4L—C4L—C5L	−130.7 (3)	O5I—C1I—C2I—O2I	−179.5 (3)
C1D—O4C—C4C—C5C	−105.4 (3)	O4J—C1I—C2I—O2I	58.3 (3)
C1D—O4C—C4C—C3C	133.2 (2)	O5I—C1I—C2I—C3I	56.0 (3)
C5I—O5I—C1I—O4J	63.7 (3)	O4J—C1I—C2I—C3I	−66.2 (3)
C5I—O5I—C1I—C2I	−57.5 (3)	C1H—O5H—C5H—C6H	−179.1 (3)
C4J—O4J—C1I—O5I	117.1 (3)	C1H—O5H—C5H—C4H	58.1 (4)
C4J—O4J—C1I—C2I	−120.6 (3)	O4H—C4H—C5H—O5H	−177.4 (2)
C1L—O5L—C5L—C6L	−179.0 (3)	C3H—C4H—C5H—O5H	−57.7 (3)
C1L—O5L—C5L—C4L	58.2 (3)	O4H—C4H—C5H—C6H	63.1 (4)
O4L—C4L—C5L—O5L	−173.3 (2)	C3H—C4H—C5H—C6H	−177.2 (3)
C3L—C4L—C5L—O5L	−55.2 (3)	C1G—O5G—C5G—C6G	−176.8 (2)
O4L—C4L—C5L—C6L	69.1 (3)	C1G—O5G—C5G—C4G	60.2 (3)
C3L—C4L—C5L—C6L	−172.8 (3)	O6G—C6G—C5G—O5G	−61.0 (3)
O4L—C4L—C3L—O3L	−68.1 (3)	O6G—C6G—C5G—C4G	59.6 (4)
C5L—C4L—C3L—O3L	173.3 (2)	O4G—C4G—C5G—O5G	−173.9 (2)
O4L—C4L—C3L—C2L	172.1 (2)	C3G—C4G—C5G—O5G	−57.1 (3)
C5L—C4L—C3L—C2L	53.5 (3)	O4G—C4G—C5G—C6G	67.5 (3)
O2L—C2L—C3L—O3L	61.8 (3)	C3G—C4G—C5G—C6G	−175.7 (3)
C1L—C2L—C3L—O3L	−172.9 (2)	O5B—C5B—C6B—O6B	72.3 (4)
O2L—C2L—C3L—C4L	−177.2 (3)	C4B—C5B—C6B—O6B	−167.9 (3)
C1L—C2L—C3L—C4L	−52.0 (3)	C4K—O4K—C1J—O5J	113.7 (3)
C5D—O5D—C1D—O4C	66.1 (3)	C4K—O4K—C1J—C2J	−126.2 (3)
C5D—O5D—C1D—C2D	−54.7 (3)	C5J—O5J—C1J—O4K	62.6 (5)
C4C—O4C—C1D—O5D	113.9 (3)	C5J—O5J—C1J—C2J	−57.8 (5)
C4C—O4C—C1D—C2D	−123.7 (3)	O2J—C2J—C1J—O4K	59.8 (4)
C1B—O5B—C5B—C6B	−177.1 (3)	C3J—C2J—C1J—O4K	−65.9 (3)
C1B—O5B—C5B—C4B	61.3 (3)	O2J—C2J—C1J—O5J	−179.2 (3)
O4B—C4B—C5B—O5B	−169.8 (2)	C3J—C2J—C1J—O5J	55.2 (4)
C3B—C4B—C5B—O5B	−54.0 (3)	C1D—O5D—C5D—C6D	−179.3 (3)
O4B—C4B—C5B—C6B	72.0 (3)	C1D—O5D—C5D—C4D	59.2 (3)
C3B—C4B—C5B—C6B	−172.1 (3)	O4D—C4D—C5D—O5D	−177.8 (2)
C1N—O4O—C4O—C3O	128.6 (3)	C3D—C4D—C5D—O5D	−59.2 (3)
C1N—O4O—C4O—C5O	−110.7 (3)	O4D—C4D—C5D—C6D	65.3 (4)
O3O—C3O—C4O—O4O	−66.9 (3)	C3D—C4D—C5D—C6D	−176.2 (3)
C2O—C3O—C4O—O4O	174.0 (2)	O5L—C5L—C6L—O6L	−57.6 (3)
O3O—C3O—C4O—C5O	173.1 (2)	C4L—C5L—C6L—O6L	62.7 (3)
C2O—C3O—C4O—C5O	53.9 (3)	C4E—O4E—C1F—O5F	106.0 (4)
O4B—C4B—C3B—O3B	−69.4 (3)	C4E—O4E—C1F—C2F	−131.7 (3)
C5B—C4B—C3B—O3B	172.9 (2)	C5F—O5F—C1F—O4E	60.7 (4)
O4B—C4B—C3B—C2B	167.2 (2)	C5F—O5F—C1F—C2F	−59.9 (4)
C5B—C4B—C3B—C2B	49.5 (3)	C1K—O5K—C5K—C6K1	−170.9 (8)
Li2—O3K—C3K—C2K	−162.5 (3)	C1K—O5K—C5K—C4K	62.3 (3)
Li2—O3K—C3K—C4K	75.9 (4)	C1K—O5K—C5K—C6K2	176.2 (9)
O2K—C2K—C3K—O3K	64.4 (3)	O4K—C4K—C5K—O5K	−173.6 (3)
C1K—C2K—C3K—O3K	−172.7 (2)	C3K—C4K—C5K—O5K	−55.4 (4)
O2K—C2K—C3K—C4K	−174.0 (2)	O4K—C4K—C5K—C6K1	62.6 (12)
C1K—C2K—C3K—C4K	−51.0 (3)	C3K—C4K—C5K—C6K1	−179.2 (12)
O4K—C4K—C3K—O3K	−67.9 (3)	O4K—C4K—C5K—C6K2	78.4 (13)
C5K—C4K—C3K—O3K	171.8 (3)	C3K—C4K—C5K—C6K2	−163.4 (13)
O4K—C4K—C3K—C2K	171.6 (2)	O5C—C5C—C6C—O6C	−65.7 (3)
C5K—C4K—C3K—C2K	51.3 (4)	C4C—C5C—C6C—O6C	53.7 (4)
C1P—O4I—C4I—C3I	131.3 (3)	O2H—C2H—C3H—O3H	55.3 (3)
C1P—O4I—C4I—C5I	−108.9 (3)	C1H—C2H—C3H—O3H	179.8 (2)
O5I—C5I—C4I—O4I	−172.8 (2)	O2H—C2H—C3H—C4H	176.5 (3)
C6I—C5I—C4I—O4I	68.5 (3)	C1H—C2H—C3H—C4H	−59.0 (3)
O5I—C5I—C4I—C3I	−55.3 (3)	O4H—C4H—C3H—O3H	−58.8 (3)
C6I—C5I—C4I—C3I	−174.0 (3)	C5H—C4H—C3H—O3H	179.8 (2)
C1A—O4H—C4H—C5H	−118.0 (3)	O4H—C4H—C3H—C2H	−179.0 (2)
C1A—O4H—C4H—C3H	121.3 (3)	C5H—C4H—C3H—C2H	59.6 (3)
C1O—O5O—C5O—C6O	−179.2 (3)	O5O—C5O—C6O—O6O	−57.4 (3)
C1O—O5O—C5O—C4O	57.5 (3)	C4O—C5O—C6O—O6O	63.1 (4)
O4O—C4O—C5O—O5O	−172.1 (2)	C5M—O5M—C1M—O4N	69.0 (4)
C3O—C4O—C5O—O5O	−54.7 (3)	C5M—O5M—C1M—C2M	−52.2 (4)
O4O—C4O—C5O—C6O	70.1 (3)	C4N—O4N—C1M—O5M	119.9 (3)
C3O—C4O—C5O—C6O	−172.4 (3)	C4N—O4N—C1M—C2M	−117.2 (3)
C1O—O4P—C4P—C5P	−128.9 (3)	O2M—C2M—C1M—O5M	177.9 (3)
C1O—O4P—C4P—C3P	110.7 (3)	C3M—C2M—C1M—O5M	51.7 (3)
C1C—O5C—C5C—C6C	−175.8 (2)	O2M—C2M—C1M—O4N	55.6 (3)
C1C—O5C—C5C—C4C	61.2 (3)	C3M—C2M—C1M—O4N	−70.6 (3)
O4C—C4C—C5C—O5C	−177.6 (2)	O3A—C3A—C2A—O2A	68.4 (3)
C3C—C4C—C5C—O5C	−57.7 (3)	C4A—C3A—C2A—O2A	−170.1 (3)
O4C—C4C—C5C—C6C	64.3 (3)	O3A—C3A—C2A—C1A	−168.0 (2)
C3C—C4C—C5C—C6C	−175.8 (3)	C4A—C3A—C2A—C1A	−46.4 (4)
O5D—C1D—C2D—O2D	176.0 (2)	C5A—O5A—C1A—O4H	63.9 (3)
O4C—C1D—C2D—O2D	53.2 (3)	C5A—O5A—C1A—C2A	−58.6 (4)
O5D—C1D—C2D—C3D	50.7 (3)	C4H—O4H—C1A—O5A	116.7 (3)
O4C—C1D—C2D—C3D	−72.0 (3)	C4H—O4H—C1A—C2A	−120.1 (3)
C5P—O5P—C1P—O4I	60.8 (3)	O2A—C2A—C1A—O5A	170.1 (3)
C5P—O5P—C1P—C2P	−59.5 (4)	C3A—C2A—C1A—O5A	47.8 (4)
C4I—O4I—C1P—O5P	110.9 (3)	O2A—C2A—C1A—O4H	47.5 (3)
C4I—O4I—C1P—C2P	−127.0 (2)	C3A—C2A—C1A—O4H	−74.8 (3)
O5P—C1P—C2P—O2P	175.7 (2)	O5M—C5M—C6M—O6M	−58.8 (4)
O4I—C1P—C2P—O2P	54.0 (3)	C4M—C5M—C6M—O6M	59.8 (4)
O5P—C1P—C2P—C3P	52.0 (3)	C5G—O5G—C1G—O4F	59.3 (3)
O4I—C1P—C2P—C3P	−69.7 (3)	C5G—O5G—C1G—C2G	−60.7 (3)
C1M—O4N—C4N—C5N	−98.6 (3)	C4F—O4F—C1G—O5G	122.8 (3)
C1M—O4N—C4N—C3N	139.2 (3)	C4F—O4F—C1G—C2G	−115.4 (3)
O4C—C4C—C3C—O3C	−62.2 (3)	O5G—C1G—C2G—O2G	179.8 (3)
C5C—C4C—C3C—O3C	176.5 (2)	O4F—C1G—C2G—O2G	59.2 (4)
O4C—C4C—C3C—C2C	177.2 (2)	O5G—C1G—C2G—C3G	57.0 (3)
C5C—C4C—C3C—C2C	55.9 (3)	O4F—C1G—C2G—C3G	−63.6 (3)
O3C—C3C—C2C—O2C	64.6 (3)	O3G—C3G—C2G—O2G	58.7 (4)
C4C—C3C—C2C—O2C	−177.3 (2)	C4G—C3G—C2G—O2G	−176.0 (3)
O3C—C3C—C2C—C1C	−172.4 (2)	O3G—C3G—C2G—C1G	−179.7 (3)
C4C—C3C—C2C—C1C	−54.3 (3)	C4G—C3G—C2G—C1G	−54.4 (3)
O2P—C2P—C3P—O3P	64.1 (3)	C1F—O5F—C5F—C6F	−169.9 (3)
C1P—C2P—C3P—O3P	−172.0 (2)	C1F—O5F—C5F—C4F	64.3 (3)
O2P—C2P—C3P—C4P	−174.1 (3)	O6F—C6F—C5F—O5F	−69.2 (3)
C1P—C2P—C3P—C4P	−50.2 (3)	O6F—C6F—C5F—C4F	51.5 (4)
O4P—C4P—C3P—O3P	−64.6 (3)	O4F—C4F—C5F—O5F	−178.5 (3)
C5P—C4P—C3P—O3P	175.2 (3)	C3F—C4F—C5F—O5F	−61.2 (4)
O4P—C4P—C3P—C2P	174.9 (2)	O4F—C4F—C5F—C6F	59.2 (4)
C5P—C4P—C3P—C2P	54.7 (3)	C3F—C4F—C5F—C6F	176.6 (3)
C1E—O4D—C4D—C3D	120.8 (3)	C1N—O5N—C5N—C6N	−178.9 (3)
C1E—O4D—C4D—C5D	−119.8 (3)	C1N—O5N—C5N—C4N	60.6 (3)
Li1—O3A—C3A—C2A	56.9 (3)	O4N—C4N—C5N—O5N	−174.2 (2)
Li1—O3A—C3A—C4A	−65.8 (3)	C3N—C4N—C5N—O5N	−54.5 (4)
O5H—C1H—C2H—O2H	−176.7 (2)	O4N—C4N—C5N—C6N	69.3 (4)
O4G—C1H—C2H—O2H	60.8 (3)	C3N—C4N—C5N—C6N	−171.0 (4)
O5H—C1H—C2H—C3H	58.5 (3)	C1A—O5A—C5A—C4A	64.4 (4)
O4G—C1H—C2H—C3H	−64.0 (3)	C1A—O5A—C5A—C6A1	−164.7 (6)
C1P—O5P—C5P—C6P	−175.3 (3)	C1A—O5A—C5A—C6A2	176.6 (6)
C1P—O5P—C5P—C4P	63.1 (4)	O4A—C4A—C5A—O5A	−177.0 (2)
O4P—C4P—C5P—O5P	−178.7 (2)	C3A—C4A—C5A—O5A	−59.2 (4)
C3P—C4P—C5P—O5P	−59.6 (3)	O4A—C4A—C5A—C6A1	55.2 (6)
O4P—C4P—C5P—C6P	63.3 (4)	C3A—C4A—C5A—C6A1	173.1 (6)
C3P—C4P—C5P—C6P	−177.6 (3)	O4A—C4A—C5A—C6A2	75.4 (7)
C1L—O4M—C4M—C3M	127.9 (3)	C3A—C4A—C5A—C6A2	−166.8 (7)
C1L—O4M—C4M—C5M	−111.2 (3)	O5P—C5P—C6P—O6P	−61.7 (4)
O3M—C3M—C4M—O4M	−60.9 (3)	C4P—C5P—C6P—O6P	56.7 (4)
C2M—C3M—C4M—O4M	179.4 (2)	C4D—O4D—C1E—O5E	108.4 (3)
O3M—C3M—C4M—C5M	177.2 (3)	C4D—O4D—C1E—C2E	−128.6 (3)
C2M—C3M—C4M—C5M	57.5 (3)	C5E—O5E—C1E—O4D	61.3 (4)
C1H—O4G—C4G—C3G	123.3 (3)	C5E—O5E—C1E—C2E	−60.6 (5)
C1H—O4G—C4G—C5G	−117.8 (3)	O4F—C4F—C3F—O3F	−63.2 (4)
O3G—C3G—C4G—O4G	−61.2 (3)	C5F—C4F—C3F—O3F	176.8 (3)
C2G—C3G—C4G—O4G	173.6 (2)	O4F—C4F—C3F—C2F	175.6 (3)
O3G—C3G—C4G—C5G	−179.5 (2)	C5F—C4F—C3F—C2F	55.6 (4)
C2G—C3G—C4G—C5G	55.3 (3)	C1F—O4E—C4E—C3E	104.8 (4)
O3O—C3O—C2O—O2O	63.5 (3)	C1F—O4E—C4E—C5E	−134.9 (3)
C4O—C3O—C2O—O2O	−175.3 (2)	Li2^ii^—O3E—C3E—C4E	−63.3 (5)
O3O—C3O—C2O—C1O	−173.5 (2)	Li2^ii^—O3E—C3E—C2E	59.6 (5)
C4O—C3O—C2O—C1O	−52.3 (3)	O4E—C4E—C3E—O3E	−67.6 (4)
C5C—O5C—C1C—O4B	58.4 (3)	C5E—C4E—C3E—O3E	174.5 (4)
C5C—O5C—C1C—C2C	−60.9 (3)	O4E—C4E—C3E—C2E	171.4 (3)
C4B—O4B—C1C—O5C	127.3 (2)	C5E—C4E—C3E—C2E	53.5 (5)
C4B—O4B—C1C—C2C	−112.3 (3)	O3E—C3E—C2E—O2E	68.0 (4)
O2C—C2C—C1C—O5C	−178.1 (2)	C4E—C3E—C2E—O2E	−169.6 (3)
C3C—C2C—C1C—O5C	57.0 (3)	O3E—C3E—C2E—C1E	−172.0 (3)
O2C—C2C—C1C—O4B	62.1 (3)	C4E—C3E—C2E—C1E	−49.5 (5)
C3C—C2C—C1C—O4B	−62.9 (3)	O4D—C1E—C2E—O2E	53.3 (4)
O4D—C4D—C3D—O3D	−62.8 (3)	O5E—C1E—C2E—O2E	175.7 (3)
C5D—C4D—C3D—O3D	177.3 (2)	O4D—C1E—C2E—C3E	−69.9 (4)
O4D—C4D—C3D—C2D	178.0 (2)	O5E—C1E—C2E—C3E	52.4 (5)
C5D—C4D—C3D—C2D	58.2 (3)	O5H—C5H—C6H—O6H2	−82.3 (5)
O2D—C2D—C3D—O3D	60.3 (3)	C4H—C5H—C6H—O6H2	38.0 (6)
C1D—C2D—C3D—O3D	−173.9 (2)	O5H—C5H—C6H—O6H1	−52.6 (6)
O2D—C2D—C3D—C4D	−179.5 (2)	C4H—C5H—C6H—O6H1	67.7 (6)
C1D—C2D—C3D—C4D	−53.7 (3)	C1E—O5E—C5E—C6E2	174.0 (7)
O4I—C4I—C3I—O3I	−64.2 (3)	C1E—O5E—C5E—C4E	61.9 (5)
C5I—C4I—C3I—O3I	176.4 (3)	C1E—O5E—C5E—C6E1	−163.6 (7)
O4I—C4I—C3I—C2I	175.6 (2)	O4E—C4E—C5E—O5E	−174.7 (3)
C5I—C4I—C3I—C2I	56.2 (3)	C3E—C4E—C5E—O5E	−57.3 (4)
O3M—C3M—C2M—O2M	61.4 (3)	O4E—C4E—C5E—C6E2	76.5 (7)
C4M—C3M—C2M—O2M	179.1 (2)	C3E—C4E—C5E—C6E2	−166.1 (7)
O3M—C3M—C2M—C1M	−173.0 (2)	O4E—C4E—C5E—C6E1	54.8 (8)
C4M—C3M—C2M—C1M	−55.3 (3)	C3E—C4E—C5E—C6E1	172.2 (8)
C4O—O4O—C1N—O5N	107.5 (3)	O3F—C3F—C2F—O2F	66.4 (4)
C4O—O4O—C1N—C2N	−131.6 (3)	C4F—C3F—C2F—O2F	−172.2 (3)
C5N—O5N—C1N—O4O	57.0 (3)	O3F—C3F—C2F—C1F	−171.0 (3)
C5N—O5N—C1N—C2N	−62.9 (3)	C4F—C3F—C2F—C1F	−49.6 (4)
C1M—O5M—C5M—C6M	174.1 (3)	O4E—C1F—C2F—O2F	53.7 (4)
C1M—O5M—C5M—C4M	55.2 (4)	O5F—C1F—C2F—O2F	175.2 (3)
O4M—C4M—C5M—O5M	−177.8 (2)	O4E—C1F—C2F—C3F	−70.6 (4)
C3M—C4M—C5M—O5M	−57.1 (3)	O5F—C1F—C2F—C3F	50.9 (4)
O4M—C4M—C5M—C6M	68.8 (3)	O5D—C5D—C6D—O6D	−62.1 (4)
C3M—C4M—C5M—C6M	−170.5 (3)	C4D—C5D—C6D—O6D	56.6 (4)
O5I—C5I—C6I—O6I	−57.5 (3)	C1J—O5J—C5J—C6J	−161.6 (5)
C4I—C5I—C6I—O6I	63.4 (3)	C1J—O5J—C5J—C4J	60.6 (6)
O3B—C3B—C2B—O2B	64.2 (3)	O4J—C4J—C5J—O5J	−178.7 (4)
C4B—C3B—C2B—O2B	−172.7 (2)	C3J—C4J—C5J—O5J	−59.9 (6)
O3B—C3B—C2B—C1B	−172.1 (2)	O4J—C4J—C5J—C6J	44.6 (7)
C4B—C3B—C2B—C1B	−49.1 (3)	C3J—C4J—C5J—C6J	163.3 (6)
O4A—C1B—C2B—O2B	57.8 (3)	O5N—C5N—C6N—O6N	56.1 (6)
O5B—C1B—C2B—O2B	179.2 (2)	C4N—C5N—C6N—O6N	174.9 (5)
O4A—C1B—C2B—C3B	−67.0 (3)	O5A—C5A—C6A2—O6A2	−56.7 (11)
O5B—C1B—C2B—C3B	54.3 (3)	C4A—C5A—C6A2—O6A2	55.8 (12)
C5O—O5O—C1O—O4P	65.8 (3)	O5A—C5A—C6A1—O6A1	61.3 (10)
C5O—O5O—C1O—C2O	−57.1 (3)	C4A—C5A—C6A1—O6A1	−173.0 (7)
C4P—O4P—C1O—O5O	108.7 (3)	O5J—C5J—C6J—O6J2	−65.2 (7)
C4P—O4P—C1O—C2O	−127.3 (2)	C4J—C5J—C6J—O6J2	68.1 (7)
O2O—C2O—C1O—O5O	178.2 (2)	O5J—C5J—C6J—O6J1	−10.6 (9)
C3O—C2O—C1O—O5O	53.4 (3)	C4J—C5J—C6J—O6J1	122.6 (8)
O2O—C2O—C1O—O4P	54.1 (3)	O5E—C5E—C6E1—O6E1	−65.4 (14)
C3O—C2O—C1O—O4P	−70.8 (3)	C4E—C5E—C6E1—O6E1	63.0 (14)
O4N—C4N—C3N—O3N	−58.9 (3)	O5E—C5E—C6E2—O6E2	75.7 (10)
C5N—C4N—C3N—O3N	178.9 (3)	C4E—C5E—C6E2—O6E2	−171.4 (8)
O4N—C4N—C3N—C2N	175.1 (2)	O5K—C5K—C6K1—O6K1	70.5 (16)
C5N—C4N—C3N—C2N	52.9 (4)	C4K—C5K—C6K1—O6K1	−166.1 (14)
C1I—O4J—C4J—C3J	127.9 (3)	O5K—C5K—C6K2—O6K2	63.8 (19)
C1I—O4J—C4J—C5J	−112.4 (4)	C4K—C5K—C6K2—O6K2	177.9 (15)
O4J—C4J—C3J—O3J	−58.8 (3)		

Symmetry codes: (i) *x*, *y*+1, *z*+1; (ii) *x*, *y*−1, *z*−1.

### Hydrogen-bond geometry (Å, º)

**Table:**

*D*—H···*A*	*D*—H	H···*A*	*D*···*A*	*D*—H···*A*
O2*K*—H171···O3*B*^iii^	0.82	1.89	2.688 (3)	165
O2*P*—H154···O4*I*	0.82	2.35	2.757 (3)	112
O2*P*—H154···O3*I*	0.82	2.07	2.799 (4)	148
O3*B*—H79···O2*C*	0.82	2.20	2.698 (3)	119
O3*C*—H83···O3*P*^ii^	0.82	1.99	2.774 (3)	161
O3*L*—H170···O2*K*	0.82	2.04	2.788 (3)	152
O3*H*—H70···O2*A*	0.82	2.07	2.533 (3)	116
O3*O*—H157···O4*O*	0.82	2.46	2.847 (3)	110
O3*O*—H157···O2*N*	0.82	2.11	2.915 (3)	168
O2*O*—H158···O3*B*^i^	0.82	1.64	2.450 (3)	167
O3*G*—H67···O2*P*^iv^	0.82	2.00	2.714 (3)	146
O3*D*—H88···O2*E*	0.82	2.15	2.702 (4)	125
O2*D*—H87···O4*C*	0.82	2.36	2.781 (3)	113
O2*D*—H87···O3*C*	0.82	2.05	2.819 (3)	156
O3*M*—H165···O3*G*^i^	0.82	2.04	2.820 (3)	160
O6*G*—H65···O6*M*^v^	0.82	1.87	2.636 (4)	154
O3*P*—H153···O4*P*	0.82	2.47	2.868 (3)	111
O3*P*—H153···O2*O*	0.82	1.82	2.611 (3)	163
O2*B*—H80···O3*O*^ii^	0.82	1.98	2.772 (3)	161
O2*L*—H169···O4*M*	0.82	2.35	2.774 (3)	113
O2*L*—H169···O3*M*	0.82	2.15	2.888 (4)	149
O2*C*—H84···O3*W*^vi^	0.82	2.42	3.116 (4)	144
O1*W*—H18*B*···O2*G*^vii^	0.86	1.93	2.781 (4)	169
O2*H*—H71···O3*H*	0.82	2.38	2.813 (3)	113
O2*H*—H71···O2*M*^ii^	0.82	2.61	3.072 (3)	118
O6*I*—H148···O6*C*^viii^	0.82	1.95	2.735 (4)	161
O2*I*—H149···O4*J*	0.82	2.43	2.818 (3)	110
O2*I*—H149···O3*J*	0.82	1.92	2.689 (4)	156
O3*J*—H176···O2*E*^i^	0.82	1.78	2.570 (4)	161
O2*A*—H73···O3*J*	0.82	1.64	2.448 (3)	167
O2*N*—H162···O2*A*^i^	0.82	1.96	2.768 (3)	170
O6*L*—H168···O7*W*^ix^	0.82	1.98	2.771 (4)	161
O6*C*—H82···O5*C*	0.82	2.43	2.818 (3)	110
O6*C*—H82···O17*W*^vi^	0.82	2.18	2.838 (6)	137
O3*I*—H150···O3*D*^i^	0.82	2.03	2.817 (3)	160
O6*F*—H61···O5*B*^iv^	0.82	1.93	2.674 (3)	151
O2*G*—H66···O3*F*	0.82	2.17	2.883 (5)	146
O11*W*—H18*C*···O2*C*^iii^	0.87	1.96	2.753 (4)	151
O11*W*—H18*D*···O16*W*	0.86	2.35	2.793 (9)	112
O2*W*—H17*A*···O8*W*	0.87	2.03	2.723 (4)	135
O2*W*—H17*B*···O10*W*	0.87	2.53	3.306 (9)	149
O2*J*—H174···O3*H*	0.86 (1)	1.85 (1)	2.677 (3)	160 (2)
O2*M*—H166···O3*N*	0.82	2.10	2.852 (4)	153
O6*B*—H78···O9*W*^x^	0.82	1.93	2.732 (4)	164
O3*E*—H59···O2*F*	0.82	2.04	2.705 (4)	137
O2*E*—H57···O3*J*^ii^	0.82	1.77	2.570 (4)	163
O6*D*—H86···O5*D*	0.82	2.38	2.789 (4)	112
O6*D*—H86···O5*W*^v^	0.82	2.15	2.767 (5)	132
O6*P*—H152···O6*B*^viii^	0.82	1.89	2.710 (4)	179
C1*L*—H109···O6*I*^iii^	0.98	2.60	3.554 (4)	165
C1*H*—H29···O3*C*^iii^	0.98	2.52	3.432 (4)	156
C4*K*—H101···O3*W*	0.98	2.61	3.409 (4)	138
C3*L*—H107···O3*F*^i^	0.98	2.64	3.515 (4)	149
C1*D*—H1···O6*G*^vi^	0.98	2.53	3.474 (4)	161
C3*K*—H100···O2*F*^i^	0.98	2.59	3.419 (5)	142
C1*P*—H133···O3*M*^vi^	0.98	2.55	3.450 (4)	152
C3*A*—H24···O2*N*^ii^	0.98	2.63	3.295 (4)	125
C4*G*—H39···O2*D*^iii^	0.98	2.60	3.423 (4)	141
C1*N*—H119···O6*W*^i^	0.98	2.47	3.420 (4)	162
C6*I*—H14*B*···O5*P*	0.97	2.52	3.349 (4)	144
C2*J*—H93···O3*H*	0.98	2.62	3.144 (4)	114
C2*I*—H143···O2*L*^vi^	0.98	2.29	3.239 (4)	162
C1*J*—H94···O18*W*	0.98	2.53	3.507 (6)	172
C6*C*—H81*B*···O5*D*	0.97	2.61	3.336 (4)	132
C6*O*—H15*B*···O5*N*	0.97	2.62	3.437 (4)	142
C6*M*—H16*D*···O5*L*	0.97	2.53	3.320 (4)	139
C2*G*—H37···O2*D*^iii^	0.98	2.41	3.300 (4)	151
O6*H*1—H69*A*···O17*W*	0.82	2.49	3.229 (14)	150
O6*H*2—H69*B*···O6*N*^v^	0.82	2.32	2.969 (9)	137
O6*N*—H160···O14*W*^i^	0.82	1.97	2.714 (10)	151
C2*F*—H46···O2*K*^ii^	0.98	2.62	3.493 (4)	148
C6*N*—H15*E*···O5*M*	0.97	2.56	3.389 (6)	143
C6*N*—H15*F*···O12*W*^i^	0.97	2.59	3.544 (15)	169
C6*A*2^a—H74^*^A^*a···O6*N*^v^	0.97	2.65	3.439 (16)	139
O6*A*2—H76*B*···O14*W*^xi^	1.19	2.11	3.276 (14)	166
O6*A*1—H76*A*···O6*N*^v^	0.82	1.94	2.755 (7)	170
C6*J*—H95*D*···O5*I*	0.97	2.53	3.424 (7)	154
C6*K*1^a—H6^*^A^*a···O5*J*	0.97	2.47	3.36 (2)	153
O6*J*1^b—H96^*^A^*b···O5*J*	0.82	2.29	2.706 (11)	112
C6*K*2^b—H6^*^AA^*b···O5*J*	0.97	2.61	3.47 (3)	147

Symmetry codes: (i) *x*, *y*+1, *z*+1; (ii) *x*, *y*−1, *z*−1; (iii) *x*, *y*+1, *z*; (iv) *x*, *y*, *z*−1; (v) *x*−1, *y*−1, *z*−1; (vi) *x*, *y*−1, *z*; (vii) *x*, *y*, *z*+1; (viii) *x*+1, *y*+1, *z*+1; (ix) *x*+1, *y*+1, *z*; (x) *x*−1, *y*−1, *z*; (xi) *x*−1, *y*, *z*.
